# Effect of an Electronic Medication Reconciliation Intervention on Adverse Drug Events: A Cluster Randomized Trial

**DOI:** 10.1001/jamanetworkopen.2019.10756

**Published:** 2019-09-20

**Authors:** Robyn Tamblyn, Michal Abrahamowicz, David L. Buckeridge, Melissa Bustillo, Alan J. Forster, Nadyne Girard, Bettina Habib, James Hanley, Allen Huang, Siyana Kurteva, Todd C. Lee, Ari N. Meguerditchian, Teresa Moraga, Aude Motulsky, Lina Petrella, Daniala L. Weir, Nancy Winslade

**Affiliations:** 1 Department of Epidemiology, Biostatistics and Occupational Health, McGill University, Montreal, Quebec, Canada; 2 Clinical and Health Informatics Research Group, McGill University, Montreal, Quebec, Canada; 3 Department of Medicine, McGill University Health Center, Montreal, Quebec, Canada; 4 Ottawa Hospital Research Institute, Ottawa, Ontario, Canada; 5 Division of Geriatric Medicine, University of Ottawa, Ottawa, Ontario, Canada; 6 McGill University Health Centre, Montreal, Quebec, Canada; 7 Centre de Recherche du Centre Hospitalier de l’Université de Montréal, School of Public Health, University of Montreal, Montreal, Quebec, Canada

## Abstract

**Importance:**

Adverse drug events (ADEs) account for up to 16% of emergency department (ED) visits and 7% of hospital admissions. Medication reconciliation is required for hospital accreditation because it can reduce medication discrepancies, but there is no evidence that reducing discrepancies reduces ADEs or other adverse outcomes.

**Objective:**

To evaluate whether electronic medication reconciliation reduces ADEs, medication discrepancies, and other adverse outcomes compared with usual care.

**Design, Setting, and Participants:**

This cluster randomized trial involved 3491 patients who were discharged from 2 medical units and 2 surgical units at the McGill University Health Centre, Montreal, Quebec, Canada, between October 2014 and November 2016. Data analysis took place from July 2017 to July 2019.

**Intervention:**

The RightRx intervention electronically retrieved community drugs from the provincial insurer and aligned them with in-hospital drugs to facilitate reconciliation and communication at care transitions.

**Main Outcomes and Measures:**

The primary outcome was ADEs in 30 days after discharge. Secondary outcomes included medication discrepancies, ED visits, hospital readmissions, and a composite outcome of ED visits, readmissions, and death up to 90 days after discharge.

**Results:**

Of 4656 eligible patients, 3567 (76.6%) consented to participate (2060 [57.8%] men; mean [SD] age, 69.8 [14.9] years). Overall, 76 patients died during the hospital stay, so 3491 patients were included in the analysis. There was no significant difference in the risk of ADEs between intervention and control groups (76 [4.6%] vs 73 [4.0%]; OR, 0.97; 95% CI, 0.33-1.48), ED visits (433 [26.2%] vs 488 [26.6%]; OR, 0.83; 95% CI, 0.36-1.42), hospital readmission (170 [10.3%] vs 261 [14.2%]; OR, 0.22; 95% CI, 0.06-1.14), or the composite outcome (447 [27.0%] vs 506 [27.6%]; OR, 0.75; 95% CI, 0.34-1.27) at 30 days. Medication discrepancies were significantly reduced in the intervention group compared with the control group (437 [26.4%] vs 1029 [56.0%]; OR, 0.24; 95% CI, 0.12-0.57). Changes made to community medications (OR, 1.05; 95% CI, 1.01-1.10) and new medications (OR, 1.09; 95% CI, 1.01-1.18) were significant risk factors for ADEs.

**Conclusions and Relevance:**

Electronic medication reconciliation reduced medication discrepancies but did not reduce ADEs or other adverse outcomes. Hospital accreditation should focus on interventions that reduce the risk of adverse events for patients with multiple changes to community medications.

**Trial Registration:**

ClinicalTrials.gov identifier: NCT01179867

## Key Points

**Question:**

Does an electronic medication reconciliation tool reduce the occurrence of adverse drug events and other adverse outcomes in the 30 days after discharge?

**Findings:**

In this cluster randomized trial that included 3491 patients discharged from 2 medical units and 2 surgical units of 1 academic hospital, electronic medication reconciliation reduced medication discrepancies but had no effect on adverse drug events (primary outcome), emergency department visits, or readmission in the 30 days after discharge.

**Meaning:**

Hospital accreditation requirements for medication reconciliation should be revised to focus on interventions that will reduce the risk of adverse events for patients with multiple changes to their community medication.

## Teaser

This cluster randomized trial evaluates whether electronic medication reconciliation reduces medication discrepancies, adverse drug events, and other adverse outcomes compared with usual care.

## Introduction

Adverse drug events (ADEs) are frequent, accounting for 8.3% to 16.2% of emergency department (ED) visits^1,2,3,4^ and up to 7% of hospital admissions^5^ at a cost of more than $5.6 million per hospital per year.^5,6^ Unintended errors in medications during transitions in care, particularly at admission, transfer, or discharge from the hospital, are thought to cause ADEs. On average, patients have between 1.2 to 5.3 discrepancies between community-based and hospital-based medications at discharge.^7^ Of these, 34% are estimated to have the potential to cause significant harm,^6^ although estimates of this potential vary from 28% to 91%.^8,9^ To mitigate the problem, the process of medication reconciliation was introduced to identify and act on discrepancies in a patient’s medication list at transitions in care, and it has quickly become an expected standard of practice. Indeed, medication reconciliation is now required for hospital accreditation in both the United States and Canada,^10,11^ and it is strongly advocated by the World Health Organization^12,13^ and the Institute for Health Improvement.^14^

Despite the obvious rationale for medication reconciliation, hospitals have struggled to achieve comprehensive adoption as the process is resource intensive and expensive. Pharmacists are the primary professionals involved in reconciliation, at an estimated cost of $3200 per 1000 prescriptions.^6,15^ A 2013 study^15^ estimated that the mean time required per patient was 92.2 minutes in geriatrics and 46.2 minutes in internal medicine at admission and 29.0 minutes in geriatrics and 19.4 minutes in internal medicine at discharge. The implementation of electronic tools to automate data retrieval and simplify the processes of reconciliation, prescribing, and communication has been shown to increase adoption and completion of the medication reconciliation process.^16,17^ There is also good evidence that medication reconciliation can reduce medication discrepancies.^6,8,9,17,18,19^ However, the missing piece in the case for medication reconciliation is evidence that a reduction in medication discrepancies will lead to a reduction in ADEs, ED visits, and readmissions.^6^ To our knowledge, this hypothesis has not been tested. To date, only interventions that combine medication review and pharmacist follow-up after discharge have been able to show a reduction in adverse events.^20,21,22^

We implemented an electronic medication reconciliation intervention (RightRx) at an academic health center and showed that it increased the rate of completion of medication reconciliation from 82.7% to 96.0% in internal medicine and from 0.7% to 80.7% in surgery.^16^ This successful implementation allowed us to test the hypothesis that medication reconciliation would not only reduce medication discrepancies for individual patients but also reduce the incidence of ADEs, ED visits, hospital readmissions, and death in the 30 days after discharge.

## Methods

The RightRx trial was conducted at the McGill University Health Centre (MUHC), a consortium of 5 tertiary hospitals for adults and children in Montreal, Quebec, Canada, with more than 1000 beds, 715 000 annual ambulatory visits, 177 000 annual ED visits, 40 000 annual admissions, and 12 000 hospital staff.^23^ The trial protocol has been previously published^24^ and is available in Supplement 1. The study was conducted in the 2 largest adult hospitals in the consortium, which account for more than 80% of adult admissions. The MUHC has a clinical information system that provides an integrated display of patient-specific hospital information including drugs, laboratory results, imaging, and prior admissions. Most clinical notes and physician orders remain paper based.

### Design, Study Population, and Randomization

To avoid contamination, a cluster randomized trial was conducted between October 2014 and November 2016 to evaluate the effectiveness of electronic medication reconciliation in reducing medication discrepancies at discharge and ADEs, ED visits, readmissions, and death in the first 30 days after discharge.^24^ All patients who were covered by provincial drug insurance, which includes seniors, welfare recipients, and those without access to employer-based private drug insurance, and who were discharged to the community or a long-term care facility from the 2 internal medicine units, the cardiac surgery unit, or the thoracic surgery unit were eligible for inclusion in the study. The eligible study population represented 55.6% of all hospitalized adult patients (4656 of 8378). Written consent from patients or their authorized proxy was received at admission, after the study was explained by the research coordinator. Patients were blinded to treatment allocation, but study staff were not. Only the first hospitalization episode was included in the analysis, even though the RightRx tool continued to be used for all hospitalized patients. Patients who died during their hospital stay were excluded from the analysis. Recruitment was based on an estimated total sample size of 3518 patients, required to ensure 80% power, at a 2-tailed α = .05, to detect an absolute reduction of 5% in ADEs, assuming 4 randomization units, with 950 patients per unit, a within-cluster correlation of 0.001, and a 15% ADE rate in the control group.^25^ The 4 units were stratified by type (ie, medicine or surgery) and hospital location (2 hospitals). All possible permutations (N = 4) of intervention and control unit assignments were created with the restriction that both trial arms should include 1 surgical and 1 medical unit and involve both hospital locations. The default random number generator in SAS version 9.4 was used to determine the final randomization of the 4 units (SAS Institute). The study was approved by the MUHC ethics committee and the Quebec privacy commissioner. Patients provided signed informed consent to participate in the trial. This study follows the Consolidated Standards of Reporting Trials (CONSORT) reporting guideline.

### Electronic Medication Reconciliation Intervention

The intervention, which is described in detail elsewhere,^16^ could be used at any point in the patient’s stay to reconcile medications at admission, transfer, and discharge. It consisted of 3 components. First, at admission, the community drug list was electronically retrieved from the provincial health insurer. The Régie de l’assurance-maladie du Québec (RAMQ), the single payer of health services in Quebec, uses its online adjudication system to connect with the 1900 pharmacies in the province to provide real-time adjudication of the coverage for all drugs dispensed to provincial beneficiaries. A secure web service was established with the RAMQ data warehouse^26^ that retrieved data on all drugs dispensed for consenting patients. Each dispensing record included the drug identification number that specifies chemical entity, manufacturer, strength, and form; the date of dispensing; quantity dispensed; duration of the prescription; name and address of the dispensing pharmacy; and the name and license number of the prescribing physician.^16^ In a prior validation study, we showed that RAMQ prescription claims achieve an accuracy of 100% for the drug dispensed and 98.5% for the date of dispensing.^27^ To prepopulate the community drug list, we included all drugs dispensed in the last 3 months to allow for nonadherence. The treatment team validated the list with the patient and added any medications not listed as well as notes about adherence, when relevant.

Second, all hospital drugs were retrieved from the hospital’s drug information system and then aligned with community medications by generic molecule, dosage, and route of administration (eFigure 1 in Supplement 2). The RightRx system is unique in that drugs are grouped by pharmacologic class and displayed in order of clinical importance rather than in alphabetic order. This reduces the cognitive load on the user while reconciling drugs such as aspirin, enoxaparin, and warfarin, which would usually appear in 3 separate areas (eFigure 1 in Supplement 2). Every morning, a list of all hospital drugs dispensed, stopped, or on hold for all patients admitted to study units was generated. This hospital list was then updated every 15 minutes with any changes made during the day. Using an action bar that enabled the user to stop, continue, or modify the dosage of each listed medication, the user could reconcile and generate a revised medication order at any point during the hospital stay or at discharge. The software was designed so that all medications had to be reconciled before an inpatient order or discharge prescription could be finalized. To facilitate communication, the user was required to use a drop-down list to select the reason for stopping a medication or changing the drug, dosage, or route of a community medication. Professional regulatory policy was used to define who could enter and save data, generate recommendations, and finalize hospital inpatient orders and discharge prescriptions. Finalized orders and discharge prescriptions were grouped by action (ie, stop, change, continue, and new prescription).

Third, the names and addresses of the community-based physicians and pharmacies who were involved in prescribing and dispensing each patient’s community medications were retrieved from the RAMQ records. The discharge prescription, including the changes made to community medications and the reasons for the changes, were faxed to each physician and pharmacy involved in the patient’s community-based care (eFigure 2 in Supplement 2). Discharge information and counseling provided to patients was the same in both groups.

### Usual Care Control

In medical units, the community drug list is generally documented at the time of admission by the pharmacist and pharmacy technicians in the ED or admitting unit. Usually, the pharmacist or technician will call the community-based pharmacy and have them fax a list of medications for a patient, a service for which community pharmacies are remunerated by the provincial insurer. Community drugs are entered into an electronic form (fillable portable document format), which is used to perform and document medication reconciliation. In surgical units, physicians and nurses in the preoperative clinic document the community drug list. There are no pharmacists assigned to the surgical units.

At discharge, the attending physician or resident uses the list of current hospital medications, with or without the community drug list, to prescribe discharge medications. Active hospital medications can be viewed by accessing the patient’s electronic record, the medication administration record, or nurse’s Kardex. The patient is provided with a paper discharge prescription to fill at the community pharmacy and may or may not receive verbal or written instructions about new medications or community medications. If the community pharmacist has questions about whether they should continue preexisting medications that are not included in the discharge prescription, they ask the patient and may call the physician or discharging unit of the hospital.

### Outcomes

#### Adverse Drug Events

We defined ADEs as an injury resulting from medical intervention related to a drug, including a failure to restart a drug that had been stopped or held during admission or an unintended therapy duplication. The Australian adverse reaction and drug event report was used to collect patient self-reported information and was administered within 25 to 30 days after discharge by telephone by a trained research assistant who was blinded to study assignment.^28^ Patients were first asked to report any new health problem or change in their condition since discharge. A review of systems was then conducted using directed probes for changes in systems-related symptoms or signs that may be drug related (eg, skin rash or cough). For positive responses, patients were asked to describe each new problem and indicate when it had started in relation to the initiation, change, or termination of drug treatment after discharge. For each patient, the medical record was abstracted to identify drugs that were started, stopped, or continued at discharge as well as acute and chronic health problems. In addition, records of all medical services claims for ED visits and hospitalizations and pharmacy claims for medications dispensed were retrieved from the RAMQ. Using the Leape-Bates ADE classification,^5,29,30^ 2 physicians at another academic health center, blinded to study assignment, independently rated the likelihood that the problem was medication-related using a visual analog probability scale (ie, very unlikely, probability 0%-15%; possible, 16%-49%; probable, 50%-84%; or very likely, ≥85%) (eFigure 3 in Supplement 2). All patients who had a positive response of a new or worsening health problem on interview or had an ED visit or readmission to hospital within 30 days after discharge were independently rated. One of us (A.H.) adjudicated any disagreement between the 2 reviewers. Agreement between reviewers was 80.5% (prevalence and bias adjusted κ,^31^ 0.61). Reviewers also rated the preventability of the ADE on a visual analog probability scale from definitely preventable to definitely not preventable using the same categories to classify probability. Agreement between reviewers on preventability was 82.7% (prevalence and bias adjusted κ, 0.65).

#### Medication Discrepancies at Discharge

The community drug list generated using the RAMQ prescription claims data for each patient was considered the criterion standard, as these records identify more than 40% more medications than are noted in the ED medical record.^32^ An *unintended error of omission* was defined as a drug that was in the community drug list but not prescribed at discharge and for which there was no documented evidence of having been stopped in the medical record. An *unintended therapy duplication* was defined as 1 drug with an active prescription in the community drug list and a second drug in the same 4-digit Anatomic Therapeutic Class (ATC)^33^ in the discharge prescription, where there was no evidence in the medical record that the community drug had been stopped or that it was to be intentionally continued. An *unintended dosage change* was defined as a 25% or greater increase or decrease in the prescribed dosage of a community medication that was not documented in the medical record as a change. To calculate the difference in dosage between community drugs and those prescribed at discharge, the strength, quantity, and duration of community-based medications were used to calculate daily dosage for all medications except creams, gels, and injectables (100 of 495 community drugs [20.2%]). The dosage for the same molecule prescribed at discharge was calculated by multiplying the dose per administration by the number of administrations per day.

#### ED Visits, Hospital Readmissions, and Death After Discharge

The secondary outcomes of ED visits, hospital readmissions, and death after discharge were measured separately and as a combined outcome in the 30 days and at 90 days after discharge in sensitivity analyses, using the RAMQ medical services claims. This approach ensured that all ED visits and readmissions were included, not just those occurring at the MUHC. This is important because ambulances transport individuals to the closest open ED or hospital, which often is not the discharging institution. Almost all hospital-based physicians in Quebec are remunerated on a fee-for-service basis,^34^ and for each medical service delivered, physicians are required to accurately record the treating establishment and the location of the service (eg, intensive care unit, ED, day hospital, or inpatient unit) because location and type of establishment determine the level of remuneration. Death was determined from 3 sources: by interview, in which information was provided by the family; by review of records of readmitted patients at the MUHC; and by evidence of a medical service claim for completion of a death certificate.

### Adjustment for Potential Confounders

Although units were assigned randomly to receive the intervention, the number of units randomized was insufficient to ensure balance in the distribution of patient characteristics between the intervention and control units. Therefore, we used propensity scores (PSs) to adjust for imbalance in potential confounders between intervention and control groups.^35^ Group assignment was the binary dependent variable in a multivariable logistic model, and predictors included patient age, sex, drug insurance status (ie, full copay, partial copay, no copay), year of admission, reason for admission (ie, first letter of the *International Statistical Classification of Diseases and Related Health Problems, Tenth Revision *[*ICD*-*10*] code), admission for an ambulatory care–sensitive condition,^36^ comorbidities (ie, presence or absence of 27 conditions included in the Charlson and Elixhauser comorbidity indices^37,38,39^), number of medical visits, number of *ICD*-*9*–coded health problems, number of community medications in each 3-digit ATC,^33^ number of prescribing physicians, number of dispensing pharmacies in the 3 months prior to admission, and number of in-hospital procedures. All variables associated with the outcome and exposure were included.^40^ Propensity score variable selection was estimated separately for the primary and secondary outcomes. In addition, we adjusted for differences in the in-hospital stay that may have had an effect on the primary and secondary outcomes, including the number of investigative and surgical procedures and the number of discharge medications. To measure each characteristic, data were retrieved from the patient’s medical record as well as from the RAMQ medical service and pharmacy claim files in the 3 months prior to admission. The resulting PS models ensured satisfactory balance, as in each quintile of the PS distribution and for each of the covariates included in the model the standardized differences between control and intervention groups were less than 0.25.^41^ Because we had preintervention data on the occurrence of ED visits and hospital admissions, we also conducted a difference-in-difference analysis for these outcomes to directly control for patient differences in background risk between the 2 trial arms. In sensitivity analyses, we adjusted directly for all potential confounders by including them as covariates in the multivariable models for each outcome instead of the PS.

### Statistical Analysis

An intention-to-treat analysis was used to determine whether electronic medication reconciliation reduced the risk of ADEs and other adverse events after discharge, using the patient as the unit of analysis. Multivariable outcome-specific logistic regression models were used to estimate the adjusted odds ratio (aOR) for the association of the intervention with ADEs. To account for clustering and to avoid the inflation of type I errors with a small number of clusters,^42,43,44^ 95% CIs for the effect of the intervention were estimated using the nonparametric 2-step cluster bootstrap method (previously developed and validated for complex analyses of clustered data)^45^ based on 10 000 bootstrap resamples. This method avoids assumptions about the covariance structure of the residuals and reliance on the asymptotic large-sample theory (eAppendix in Supplement 2). The same approach was used to assess the potential effects of the intervention on medication discrepancies.

For ED visits, hospital readmission, and the combined outcome, we conducted a difference-in-differences analysis.^46^ These analyses pooled data on all consenting patients discharged from the study units (clusters) in the preintervention and postintervention periods. A binary variable indicating the period (ie, before vs after the intervention) and its interaction with the intervention were both added to the outcome-specific multivariable models. The period × intervention interaction was then estimated and tested to assess whether there was a greater reduction in ED visits and hospital admissions among the intervention group compared with the control group in the postintervention period relative to the preintervention period. For primary and secondary outcomes, we adjusted for baseline differences between patients in the usual care and intervention arms by adjusting the multivariable models for the PSs, the number of procedures, and the number of discharge medications. In sensitivity analyses, we assessed whether the inclusion of all individual covariates in the model, instead of the PS, influenced the estimated intervention effect.

It has been suggested that medication reconciliation should primarily focus on groups with higher risk.^18^ Therefore, to determine if the effect of the intervention was modified by patient characteristics that have been associated with a higher risk of adverse events (ie, age, number of medication changes, and number of discharge medications^25,47,48^), we included the respective 2-way interaction terms in the multivariable logistic model and assessed their statistical significance using 2-step cluster bootstrap-based 95% CIs. To understand the association of the expected outcome of medication reconciliation (ie, a reduction in medication discrepancies) with ADEs, we estimated the association of ADEs with medication discrepancies (overall and by type), with the number of medications at discharge, and with the number and type of changes made to community medications. As it has been postulated that a 1-month follow-up may not be long enough to see the beneficial effects of medication reconciliation on adverse events,^6^ we used the already described methods in sensitivity analysis to assess the potential effects of medication reconciliation on the risk of adverse events observed until up to 90 days after discharge.

Statistical analyses were conducted using SAS version 9.4 statistical software (SAS Institute). Statistical significance was set at *P* = .05, and all tests were 2-tailed.

## Results

Overall, 8378 patients were admitted to study units between October 2014 and November 2016, and 3722 were excluded. The main reasons for ineligibility were the absence of RAMQ drug insurance coverage (1930 [23.0%]) or an in-hospital transfer to a nonstudy unit (1468 [17.5%]) (Figure). Of 4656 eligible patients, 3567 (76.6%) consented to participate, of whom 2060 (57.8%) were men, and the mean (SD) age was 69.8 (14.9) years (Table 1, Figure). Among the 2453 eligible patients in control units and 2203 in intervention units, an equivalent proportion consented to participate in the study. Overall, 76 patients died during the hospital stay, 41 (2.2%) in the control group and 35 (2.1%) in the intervention group. The remaining 3491 patients (1836 patients in the control group and 1655 patients in the intervention group) were included in the analysis.

**Figure.**
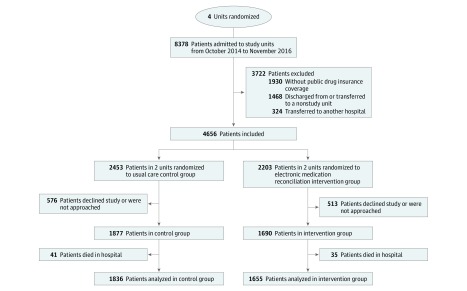
Flow Diagram of Patients Included in the RightRx Trial

**Table 1.** Characteristics of the 3567 Patients Enrolled in the Intervention and Control Units

Patient Characteristic	No. (%)		
	Overall (N = 3567)	Intervention (n = 1690)	Control (n = 1877)
Demographic characteristics			
Age, mean (SD), y	69.8 (14.9)	70.6 (13.6)	69.0 (15.9)
Men	2060 (57.8)	1048 (62.0)	1012 (53.9)
Copayment status			
Full payment	2101 (58.9)	1027 (60.8)	1074 (57.2)
Partial payment	812 (22.8)	381 (22.5)	431 (23.0)
Free	654 (18.3)	282 (16.7)	372 (19.8)
Comorbidities			
Myocardial infarction	206 (5.8)	175 (10.4)	31 (1.7)
Congestive heart failure	575 (16.1)	326 (19.3)	249 (13.3)
Peripheral vascular disease	243 (6.8)	120 (7.1)	123 (6.6)
Cerebrovascular disease	224 (6.3)	106 (6.3)	118 (6.3)
Chronic pulmonary disease	555 (15.6)	210 (12.4)	345 (18.4)
Connective tissue disease/rheumatic	114 (3.2)	66 (3.9)	48 (2.6)
Dementia	161 (4.5)	71 (4.2)	90 (4.8)
Peptic ulcer disease	52 (1.5)	19 (1.1)	33 (1.8)
Mild liver disease	167 (4.7)	56 (3.3)	111 (5.9)
Diabetes without chronic complications	785 (22.0)	402 (23.8)	383 (20.4)
Diabetes with chronic complications	66 (1.9)	25 (1.5)	41 (2.2)
Paraplegia or hemiplegia	27 (0.8)	9 (0.5)	18 (1.0)
Renal disease	344 (9.6)	157 (9.3)	187 (10.0)
Cancer	1197 (33.6)	297 (17.6)	900 (47.9)
Moderate and severe liver disease	16 (0.5)	5 (0.3)	11 (0.6)
Metastatic carcinoma	363 (10.2)	66 (3.9)	297 (15.8)
HIV/AIDS	39 (1.1)	11 (0.7)	28 (1.5)
Charlson Comorbidity Index score, mean (SD)	2.5 (2.7)	1.8 (2.1)	3.1 (2.9)
Health care use 3 mo before admission, mean (SD), No.			
Ambulatory medical visits	4.4 (5.2)	3.7 (5.2)	5.0 (5.2)
Emergency department visits	1.4 (2.1)	1.3 (1.8)	1.5 (2.3)
Hospitalizations	0.3 (0.6)	0.3 (0.6)	0.2 (0.6)
Medication use at admission, mean (SD), No.			
Community medications	7.9 (5.8)	7.6 (5.4)	8.2 (6.0)
Pharmacies	1.1 (0.6)	1.1 (0.6)	1.1 (0.6)
Prescribing physicians	2.8 (2.2)	2.7 (2.0)	3.0 (2.3)
Index hospitalization			
Admitted from			
Community	3139 (88.0)	1382 (81.8)	1757 (93.6)
Another hospital	407 (11.4)	298 (8.4)	109 (3.1)
Long-term care	16 (0.5)	8 (0.5)	8 (0.5)
Rehabilitation hospital	5 (0.2)	2 (0.1)	3 (0.2)
Medications prescribed at discharge, mean (SD), No.	10.3 (5.3)	11.5 (4.8)	9.1 (5.5)
Changes in medications at discharge, mean (SD), No.	6.3 (4.6)	8.4 (5.0)	4.4 (3.3)
New medications, mean (SD), No.	4.0 (3.2)	5.3 (3.3)	2.9 (2.5)
Stopped medications, mean (SD). No.	1.6 (2.4)	2.3 (2.8)	0.9 (1.6)
Dosage changes, mean (SD), No.	0.7 (1.0)	0.8 (1.1)	0.6 (0.9)

The intervention and control groups were similar in comorbidity, health care, and medication use with a number of exceptions (Table 1). In the intervention group, there was a higher proportion of men (1048 [62.0%] vs 1012 [53.9%]) and patients with myocardial infarction (175 [10.4%] vs 31 [1.7%]). In the control group, there was a higher proportion of patients with cancer (900 [47.9%] vs 297 [17.6%]), and the mean (SD) Charlson Comorbidity Index score was higher (3.1 [2.9] vs 1.8 [2.1]). These differences were predominantly associated with the specialized nature of the surgical units in the intervention group (cardiac) and control group (thoracic). A slightly higher mean (SD) number of drugs was prescribed at discharge in the intervention group (11.5 [4.8] vs 9.1 [5.5]), particularly the number of new medications (5.3 [3.3] vs 2.9 [2.5]). In sensitivity analysis, the models that adjusted for all individual covariates improved the fit over the model that adjusted for the PSs, so all reported results are based on models that adjusted for all covariates.

Overall, 149 patients (4.3%) experienced an ADE during the first 30 days after discharge; 114 ADEs (76.5%) were considered definitely preventable and 30 (20.1%) probably preventable (Table 2). There was no significant difference in ADE rates in the intervention group compared with the control group (76 [4.6%] vs 73 [4.0%]; aOR, 0.97; 95% CI, 0.33-1.48), even when we limited the outcome to definitely preventable ADEs (58 [3.5%] vs 56 [3.1%]; aOR, 0.85; 95% CI, 0.28-1.31) and probably preventable ADEs (16 [1.0%] vs 14 [0.8%]; aOR, 1.45; 95% CI, 0.20-12.19) (Table 2). The effect of the intervention on ADEs was not modified by age, the number of discharge medications, or changes in community medications.

**Table 2.** Primary and Process Outcomes in the 30 Days After Discharge

Outcome	No. (%)			OR (95% CI)^a^
	Overall (n = 3491)	Intervention (n = 1655)	Control (n = 1836)	
**Primary Outcome**				
Adverse drug event	149 (4.3)	76 (4.6)	73 (4.0)	0.97 (0.33-1.48)
Definitely preventable	114 (3.3)	58 (3.5)	56 (3.1)	0.85 (0.28-1.31)
Probably preventable	30 (0.9)	16 (1.0)	14 (0.8)	1.45 (0.20-12.19)
Probably or definitely not preventable	5 (0.1)	2 (0.1)	3 (0.2)	NA^b^
**Process Outcomes**				
Any medication discrepancy	1466 (42.0)	437 (26.4)	1029 (56.0)	0.24 (0.12-0.57)
Error of omission^c^	919 (26.3)	131 (7.9)	788 (42.9)	0.08 (0.02-0.41)
Therapy duplication^d^	225 (6.4)	39 (2.4)	186 (10.1)	0.10 (0.00-0.34)
Unintended dosage change^e^	742 (21.3)	328 (19.8)	414 (22.5)	0.75 (0.49-1.81)

Abbreviations: NA, not applicable; OR, odds ratio.

^a^ Odds ratios for adverse drug events were obtained from models that adjusted for all covariates significantly associated with the outcome: age, sex, number of in-hospital procedures, number of drugs at discharge, number of chronic conditions, cancer, hypertension, multiple sclerosis, cardiac valve disease, schizophrenia, number of visits 3 months prior to admission, number of drugs at admission, admission from a rehabilitation hospital, diuretics prescribed at discharge (yes or no), other therapeutic categories prescribed at discharge (yes or no), and admission through the emergency department. Odds ratio for medication discrepancies were obtained from models that adjusted for all covariates significantly associated with the outcome: age, sex, number of prescribing physicians, number of pharmacies, number of community medications, number of ambulatory visits before admission, admission from a rehabilitation hospital, admission through the emergency department, number of drugs at discharge, number of in-hospital procedures, cancer, hypertension, heart failure, peripheral vascular disease, cerebrovascular disease, osteoporosis, epilepsy, diabetes, chronic obstructive pulmonary disease, depression, ulcers, rheumatoid arthritis, and community medications in the following classes: antiprotozoals, antivirals, thyroid therapy, hormonal therapy, psycholeptics, antipruritics, immunostimulants, drugs for obstructive airway disease, and general nutrients. The nonparametric 2-step cluster bootstrap method was used to estimate 95% CIs.

^b^ Odds ratio unavailable because of the small frequency of this response.

^c^ Defined as a drug that was in the community drug list that was not prescribed at discharge and for which there was no documented evidence of having been stopped in the medical record.

^d^ Defined as 1 drug with an active prescription in the community drug list and a second drug in the same 4-digit Anatomic Therapeutic Class^33^ in the discharge prescription, where there was no evidence in the medical record that the community drug had been stopped or that it was to be intentionally continued.

^e^ Defined as a 25% or greater increase or decrease in the prescribed dosage of a community medication that was not documented in the medical record as a change.

The RightRx system was used to complete medication reconciliation at discharge for 1464 of 1655 patients (88.5%), 763 of 799 (95.5%) in internal medicine, and 701 of 856 (81.9%) in cardiac surgery. Although there was no difference in ADEs, the RightRx intervention was associated with a significant reduction in the proportion of patients with at least 1 medication discrepancy (437 [26.4%] vs 1029 [56.0%]; aOR, 0.24; 95% CI, 0.12-0.57) (Table 2). This included significant reductions in errors of omission (131 [7.9%] vs 788 [42.9%]; aOR, 0.08; 95% CI 0.02-0.41) and therapy duplications (39 [2.4%] vs 186 [10.1%]; aOR, 0.10; 95% CI, 0.00-0.34) but not unintended dosage changes (328 [19.8%] vs 414 [22.5%]; aOR, 0.75; 95% CI, 0.49-1.81). The effect of the intervention was significantly modified by the number of discharge medications and patient age. For example, for patients aged 60 years and discharged with 7 medications, the intervention reduced the odds of a discrepancy by 75% (aOR, 0.25; 95% CI, 0.07-0.53) compared with patients aged 60 years and discharged with 14 medications, for whom it reduced the odds of a discrepancy by 84% (aOR, 0.16, 95% CI, 0.02-0.40), and patients aged 80 years with 7 discharge medications, for whom it reduced the odds of a discrepancy by 82% (aOR, 0.18; 95% CI, 0.05-0.45).

In the 30 days after discharge, 921 patients (25.8%) had an ED visit, 431 (12.3%) were readmitted to the hospital, 86 (2.5%) died, and 953 (27.3%) had at least 1 of these adverse outcomes (Table 3). In the 30 days prior to the index admission, the proportion of patients with an ED visit or hospital admission was higher in the intervention group than in the control group (ED visit: 714 [43.1%] vs 711 [38.7%]; hospital admission: 369 [22.3%] vs 164 [8.9%]). The difference-in-difference analysis showed an additional reduction from the preintervention to postintervention period in the intervention group compared with concurrent changes (ie, before to after) in the control group of 17% for the period × intervention interaction in ED visits (433 [26.2%] vs 488 [26.6%]; aOR, 0.83; 95% CI, 0.36-1.42), 78% in hospital admission (170 [10.3%] vs 261 [14.2%]; aOR, 0.22; 95% CI, 0.06-1.14), and 25% in the composite outcome (447 [27.0%] vs 506 [27.6%]; aOR, 0.75; 95% CI, 0.34-1.27) in the 30 days after discharge. However, after adjustment for clustering, these reductions were not statistically significant, as the 2-step cluster bootstrap-based 95% CIs for the interaction included 0. In sensitivity analysis, extending the follow-up to 90 days did not result in a statistically significant reduction in these adverse outcomes. While the interaction of the number of changes made to medications and the intervention showed a pattern of increasing benefit of the intervention with each additional change to community medications, this interaction was not statistically significant.

**Table 3.** Secondary Outcomes in the 30 Days and 90 Days After Discharge

Outcome	No. (%)						OR (95% CI)^a^
	Before Trial			After Trial			
	Overall (N = 3491)	Intervention (n = 1655)	Control (n = 1836)	Overall (N = 3491)	Intervention (n = 1655)	Control (n = 1836)	
**Secondary Outcomes at 30 d After Discharge**							
ED visits	1425 (40.8)	714 (43.1)	711 (38.7)	921 (25.8)	433 (26.2)	488 (26.6)	0.83 (0.36-1.42)
Hospital readmissions	533 (15.3)	369 (22.3)	164 (8.9)	431 (12.3)	170 (10.3)	261 (14.2)	0.22 (0.06-1.14)
ED visits, readmissions, or death	1524 (43.7)	782 (47.3)	742 (40.4)	953 (27.3)	447 (27.0)	506 (27.6)	0.75 (0.34-1.27)
**Secondary Outcomes at 90 d After Discharge**							
ED visits	1872 (53.6)	877 (53.0)	995 (54.2)	1518 (43.5)	694 (41.9)	824 (45.0)	0.94 (0.70-1.22)
Hospital readmissions	797 (22.8)	463 (28.0)	334 (18.2)	725 (20.8)	292 (17.6)	433 (23.6)	0.37 (0.11-1.40)
ED visits, admissions, or death	1984 (56.8)	942 (56.9)	1042 (56.8)	1600 (45.8)	728 (44.0)	872 (47.5)	0.87 (0.62-1.18)

Abbreviations: ED, emergency department; OR, odds ratio.

^a^ Odds ratios were adjusted for all covariates significantly associated with the outcome: age, sex, number of prescribing physicians, admission through the ED, number of chronic conditions, number of discharge medications, number of in-hospital procedures, Charlson Comorbidity Index score, heart failure, chronic obstructive pulmonary disease, depression, ischemic heart disease, cerebrovascular disease, epilepsy, Alzheimer disease, hypertension, peripheral vascular disease, osteoporosis, lymphoma, and the use of medications in the following therapeutic classes: muscle relaxants, drugs for diabetes, general nutrients, antiemetics, immunosuppressants, β-blockers, laxatives, and other therapeutic classes. The nonparametric 2-step cluster bootstrap method was used to estimate 95% CIs.

We examined whether medication discrepancies were associated with ADEs, as the intended impact of medication reconciliation is to prevent these discrepancies from occurring as they are thought to cause harm. An increased number of medication discrepancies was not significantly associated with an increased ADE risk (aOR, 1.08; 95% CI, 0.94-1.22) (Table 4). Of all discrepancies, the number of unintended dosage changes appeared to be the most important; however, it was not significantly associated with an increased ADE risk (aOR, 1.17; 95% CI, 0.79-1.56). Although the number of discharge medications was not associated with the risk of an ADE after discharge, the number of changes made to community medications and the number of new medications that were added to a patient’s therapeutic regimen were both significant risk factors for ADEs. For every change made in community medication, the risk of an ADE increased by 5% (aOR, 1.05; 95% CI, 1.01-1.10) and for every new medication added, the risk of an ADE increased by 9% (aOR, 1.09; 95% CI, 1.01-1.18).

**Table 4.** Medication-Related Discharge Characteristics Associated With Adverse Drug Events

Medication-Related Discharge Characteristic	Mean (SD), No.			OR (95% CI)^a^
	Overall (N = 3491)	Adverse Drug Event (n = 149)	No Adverse Drug Event (n = 3342)	
**Medication Discrepancies per Patient**				
Medication discrepancies	1.2 (2.1)	1.39 (2.2)	1.17 (2.1)	1.08 (0.94-1.22)
Omissions	0.8 (1.9)	0.87 (1.9)	0.80 (1.9)	1.07 (0.87-1.22)
Therapy duplications	0.1 (0.3)	0.07 (0.3)	0.08 (0.3)	0.97 (0.31-1.79)
Unintended dosage changes	0.3 (0.7)	0.45 (0.9)	0.29 (0.7)	1.17 (0.79-1.56)
**Discharge Medications per Patient**				
Discharge medications	10.3 (5.3)	11.5 (5.4)	10.2 (5.3)	1.03 (0.99-1.07)
Changes in community medications	6.4 (4.6)	7.5 (5.1)	6.3 (4.6)	1.05 (1.01-1.10)
New medications	4.1 (3.1)	4.5 (3.5)	4.1 (3.1)	1.09 (1.01-1.18)
Stopped medications	1.6 (2.4)	2.1 (2.5)	1.6 (2.4)	1.04 (0.96-1.11)
Dosage changes	0.7 (1.0)	0.9 (1.2)	0.7 (1.0)	1.13 (0.91-1.40)
Dosage increases	0.3 (0.6)	0.4 (0.8)	0.3 (0.6)	1.20 (0.82-1.71)
Dosage decreases	0.4 (0.8)	0.5 (0.9)	0.4 (0.8)	1.07 (0.84-1.30)

Abbreviation: OR, odds ratio.

^a^ Discharge medication characteristics were estimated in a separate model from medication discrepancies to avoid multicollinearity. The models adjusted for all covariates significantly associated with adverse drug events: age, sex, number of in-hospital procedures, number of drugs at discharge, number of chronic conditions, cancer, hypertension, multiple sclerosis, cardiac valve disease, schizophrenia, number of visits 3 months prior to admission, number of drugs at admission, admission from a rehabilitation hospital, diuretics prescribed at discharge (yes or no), other therapeutic categories prescribed at discharge (yes or no), and admission through the emergency department.

## Discussion

We found that electronically enabled medication reconciliation was successfully used in more than 80% of discharges but had no significant effect on ADEs at 30 days after discharge or on ED visits or hospital readmissions. However, it produced a substantial and significant reduction in unintended discrepancies between community and hospital medications.

While the design of the RightRx system was effective in virtually eliminating medication discrepancies, it did not lead to a reduction in ADEs. Although the rate of ADEs was lower than expected in initial sample size calculations, we had a power of 80% to detect absolute differences as small as 2.5% between intervention and control groups with an intracluster correlation of 0.001 found in our study. It is possible that medication discrepancies at discharge were corrected by either the community pharmacist or physician before they caused potential harm. However, it has been suggested that many medication discrepancies have limited potential to cause harm.^6^ Our results support this contention, as we found no significant association of the number or type of discrepancies with ADEs. However, other aspects of the patient’s discharge medication are important risk factors for ADEs, particularly the number of changes to community medications and the number of new medications. Others have noted that the greatest risk of ADEs occurs when starting or changing medications.^25,49,50^ Because many changes are made to community medications during hospitalization—a mean of 6.3 per patient in the units involved in this study—close follow-up in the immediate postdischarge period to address medication-related adverse effects may be beneficial. Approximately 44% of patients in this study did not adhere to at least 1 of the changes made to their medication after discharge (D.L.W., unpublished data, June 2019). This may be why the only studies that have shown a reduction in ADEs with medication reconciliation are those that included medication review and follow-up by a pharmacist after discharge as part of the intervention.^21,51^ Finally, we may have measured adverse drug events too early. The possibility that the potential harm caused by medication discrepancies, particularly errors of omission, may not be immediate was raised by Kwan et al.^6^ However, extending the follow-up window to 90 days after discharge did not show any incrementally improved benefit of the intervention, possibly because discrepancies were corrected by the patient’s primary care team. These hypotheses would need to be investigated in future studies.

Hospital accreditation requirements in Canada and the United States recommend that medication reconciliation be carried out for all patients at admission, transfer, and discharge. This standard has been difficult to achieve because of the resource requirements, and many hospitals have therefore focused their efforts on more complex patients with multiple medications.^52^ Our results do not provide empirical support for hospital accreditation requirements that are based on the contention that medication reconciliation will reduce discrepancies that lead to ADEs, ED visits, hospital readmission, or death after discharge. These were common events in our study population, with 4.3% of patients having an ADE and nearly 1 of 3 patients returning to the ED, being readmitted, or dying in the 30 days after discharge. Our results do support the contention that interventions to reduce ADEs should be targeted to patients with high risk who have multiple changes made to their community medications and many new medications added to their therapeutic regimen.

### Strengths and Limitations

There are a number of strengths in this study. First, we integrated population-wide insurance data to provide comprehensive information on community-based medications, ED visits, hospital readmissions, and death. While we do not know what medications were prescribed, only what was dispensed, the community-based medication data increased the value and adoption rate for the intervention by automating the input of community-based medications. They also provided comparable unbiased information for assessing outcomes of patients discharged from intervention and control units. Second, we achieved high rates of medication reconciliation in both intervention units as well as high and equivalent rates of patient consent, enabling us to generate robust evidence about the impact of medication reconciliation on adverse events in an intention-to-treat analysis.

This study has limitations. The limitations of our study are primarily related to generalizability, as the cluster randomized trial took place at 1 academic health center and 4 medical or surgical units. Moreover, randomization of only 4 units would not ensure balance in patient characteristics between intervention and control groups, although the difference-in-difference analysis minimized the potential effects of unmeasured confounding. In addition, the number of unintentional discrepancies in the control units may be overestimated as we relied on health record documentation, which may be incomplete, to determine if there was a rationale for omissions, dosage changes, and therapy duplications, whereas the RightRx intervention required all discrepancies to be addressed by the physician to create the discharge prescription.

## Conclusions

This study found that electronic medication reconciliation reduced medication discrepancies, but it did not have the expected effect of reducing ADEs. Hospital accreditation should focus on interventions that will reduce the risk of adverse events for patients with multiple changes to their community medication.

## Notes

Supplement 1. **Trial Protocol**

Supplement 2. **eAppendix.** Analysis **eFigure 1.** Screenshot of the RightRx User Interface at Discharge **eFigure 2.** Discharge Prescription **eFigure 3.** ADE Adjudication Forms **eReferences.**

Supplement 3. **Data Sharing Statement**
