# Supplementary material for: Effect of an Electronic Medication Reconciliation Intervention on Adverse Drug Events: A Cluster Randomized Trial
Source: JAMA Netw Open. 2019 Sep 20;2(9):e1910756. doi: 10.1001/jamanetworkopen.2019.10756 (PMC6755531; doi:10.1001/jamanetworkopen.2019.10756)
Supplement: Supplement 1. — Trial Protocol [file jamanetwopen-2-e1910756-s001.pdf]

Using Novel Canadian Resources to Improve Medication Reconciliation at Discharge

PROTOCOL

Purpose

The purpose of this research is to reduce the risk of preventable adverse drug events and hospital re-admissions post discharge by testing novel strategies to improve the transmission and reconciliation of medication information at discharge from hospital.

Preventable Adverse Drug Events

Up to a quarter of admissions to acute care hospitals are related to adverse drug events (ADE). ADEs are the 6th leading cause of death at a cost over $5.6 million per hospital per year [1]. Fortunately, at least 58% of these ADE's are preventable, as they result from incomplete drug information, prescribing or dispensing errors, and overuse or underuse of medications[2]. The convergence of increasingly complex care plans, an aging patient population, and multiple transfers between care settings (long term, acute care, community) account for most of the preventable errors that ultimately result in ADEs. In fact, at admission, 60%-70% of medication histories contain at least one error, the majority of which are errors in omitted medication, and 37% have the potential to cause harm[3-8]. Failure to obtain a complete profile of pre-admission medication is responsible for 72% of potential adverse drug events after discharge[2,6]. An estimated 19% to 23% of patients will have an adverse event within 30 days of discharge[9,10], 14.3% will be readmitted[11], and 70% of these events will be related to prescription medication[9,10].

Medication Reconciliation at Discharge

Reconciliation of community-based medications with changes during hospitalization is a critical requirement to reduce the risk of preventable adverse drug-related events during transitions in care. Its importance is evident in hospital accreditation standards in Canada[2] and the United States, which now require implementation of a method for medication reconciliation[12-15]. Essentially, medication reconciliation at discharge involves two elements: 1) obtaining the community-based drug history of current active medication, and determining which drugs have been stopped, changed, or added during the hospital stay, and 2) communicating information on changes in community-based medication made during hospitalization to the responsible pharmacists and physicians at discharge. In the past few years, considerable experience has accumulated on the challenges of implementing medication reconciliation in a variety of clinical areas and patient populations [7,8,16-31]. A number of critical challenges have been identified that limit effective implementation, particularly in transitions between community and hospital settings.

Challenge #1 Obtaining an accurate community-based medication list

Difficulty in obtaining accurate information about the community–based drug list is one of the greatest challenges in medication reconciliation[32]. In a recent survey of hospital staff, respondents estimated that 87% of admitted patients did not know which medications they took, 80% of the time medication information was not available from alternate sources such as relatives or community-based care providers, and in 63% of admissions, hospital staff were unable to access community-based records [32]. As a result, 46%-67% of unintended discrepancies in medication reconciliation are omitted medications, that is medications that were taken in the community but were neither prescribed at admission nor reconciled at discharge[3-8,18]. The most commonly omitted medications are cardiovascular drugs, pain medications, anti-infectious medications, and central nervous system medications such as anti-depressants and sleeping pills [33]. Overall, 23% to 37% of unintended discrepancies between community and hospital medication are considered clinically significant, meaning that there is substantial potential to cause harm[3-8,18].
An increasing number of hospitals are employing pharmacists in the emergency department and inpatient units to obtain a complete history of community-based medications[12-14,18-20,22,34-40]. Pharmacists have been shown to be more effective than nurses or medical staff in obtaining an accurate medication history, reducing errors from 323 to 86 per 1,000 prescription orders compared to nurse-taken histories where errors

---

[1] www.ahrq.gov/qual/aderia/aderia.htm
[2] www.accreditation.ca/uploadedFiles/Knowledge_Exchange/Patient_Safety/Required_Organizational_Practices

were reduced to only 157 per 1,000 [33]. The superiority of pharmacists in medication history-taking may be related to two aspects of care. First, pharmacists spend an average of 12.9 minutes per patient to take a community-based medication history, 2-3 times longer than medical or nursing staff[41]. Second, pharmacists dispense medication and in general are much more knowledgeable about medication characteristics. This expertise may be particularly useful when patients are attempting to recall their medication, as most patients remember their medications by the colour, shape and general purpose of the pill—*"I take a round orange pill every morning for my blood pressure" (translation: Apo-Hydrochlorithizaide 12.5 mg. OD)*[3][29,42]. Pharmacists may be more likely to identify these medications than medical and nursing staff, who know the name but not usually the colour and shape of the pill. Indeed, a recent pilot study performed in a U.S. Veteran's Affairs hospital found that the integration of pill image files with medication lists was a useful approach to verify current use with patients[29].

Although pharmacist deployment in clinical care areas is considered a cost-effective investment in preventing medication errors,[43] pharmacists are conventionally not available on weekends, evenings and nights, nor are community-based pharmacies or office-based practices usually open to transmit information about community-based medications by fax or telephone. As such, recent research has shown that unintended errors in reconciling community and admission medications at discharge are more likely to occur at night-time admission, particularly for elderly patients and those using >4 medications [28].

New initiatives have been undertaken to use electronic medical records to access information about the community drug profile[8,30,31,44]. Brigham and Women's Hospital in Boston has shown that retrieval from electronic medical records can identify 65% of current medications[30]. The major limitations of using medication lists in electronic medical records is that many of the listed medications (up to 70%) are no longer being used by the patient as medication lists become out-of-date, and 15.5% of current medications are not listed in the electronic medical record[44]. Moreover, low adoption rates of electronic medical records in Canada and the United States, means that retrieval of community medications from electronic medical records may be a mid to long term solution for effective medication reconciliation[45].

In contrast, almost all pharmacies in North America have been computerized so that they can manage the on-line adjudication processes of public and private drug insurance programs[46]. Prior research has shown that records of dispensed prescriptions can be used to accurately measure medication adherence[47-49]. A recent study from the Netherlands also suggests that community pharmacy records can identify up to 97.6% of community-based medications accurately[8]. Although it represents a promising approach, the utility of community-based pharmacy records for medication reconciliation at hospital discharge has not been formally assessed.

Challenge #2 Ensuring Medication Reconciliation is conducted for all Patients at Risk

In compliance with accreditation standards, most hospitals have instituted a paper-based medication reconciliation process. However, adherence is poor, with medication reconciliation generally conducted in less than 20% of patients at risk[12-14,18-20,22,34-40]. This low rate of utilization persists even when staff workload is reduced by an electronic "copy and paste" process that eliminates the need to first document the community-based medication list and then re-transcribe the list for the hospital medication order[28,50]. One of the main barriers is the time and resources required for data collection (community drug list determination), particularly in emergency departments (ED) from where most patients are admitted. For a typical ED with 50,000 visits per year, it is estimated that an additional 2,900 hours of nursing time, and 8,750 hours of pharmacist time would be required (an added cost of $349,500 at $30/hour) to complete the admission medication reconciliation for the 35% of patient visits where it is required[51]. Moreover, 20% of patients die or are discharged before complete information can be obtained about the community drug list[51]!

Overcoming inefficiencies in obtaining the community drug list appears to be essential to improve adherence. For example, when Brigham and Women's Hospital established a prototype medication reconciliation module that integrated data from the ambulatory electronic medical record and discharge

---

[3] Mrs X, emergency department visit medication history by Mme. P (clinical pharmacist), May 10th, 2009

medication orders, they improved adherence to 68.7%, as the majority of physicians could reduce the time to complete the process by 10 minutes. Even higher rates of adherence—from 20% to 90% at admission and 95% at discharge—were achieved at Bellevue Hospital in New York, when admission and discharge orders were blocked until the medication reconciliation module was completed[50].However, this option is only possible in hospitals who have successfully implemented computerized physician order-entry, which represents less than 20% of hospitals in the United States and even fewer in Canada[52-54].

Challenge #3 Communicating Drug/Dose Changes at Discharge to Community-based Prescribing Physicians and Dispensing Pharmacists

A substantial proportion of adverse drug-related events occur in hospitalized patients shortly after discharge[9,10]. It is estimated that 72% of medication reconciliation errors at discharge are due to an incomplete preadmission community drug list, while 26% are due to failures in reconciling the medication history or changes made during the hospital stay with discharge orders[6]. During hospitalization 31% of patients will have changes made in the dose and frequency of medication, 9% will have a medication added or substituted and 4.1% to 8% will have a medication stopped[6,55]. At the present time, there is no timely and effective mechanism of communicating these changes in medication to the community-based prescribing physician(s) and dispensing pharmacist(s). Most patients fill their discharge medication prescription within the first few days after hospital discharge[56], far before the discharge summary that summarizes the reasons for hospitalization and changes in medical management has been dictated or transmitted. Indeed, in the majority of admissions, the community-based care team does not receive critical information on the patient's health status and modified treatment plan post-discharge[57]. As a result, the patient's community-based pharmacist(s) needs to determine whether remaining refills on community-based drugs are to be added to the discharge prescription or stopped; and whether the dose prescribed on a discharge medication is to be added or replace the existing pre-admission medication dose. As the community-based profile is typically incomplete, these issues are usually not addressed in the discharge prescription. To add to the challenges of discharge reconciliation, 70% of elderly patients who use many medications are under the care of a number of prescribing physicians and over 40%of patients will use more than one dispensing pharmacy[58,59]. For all of these reasons, it is not surprising that 17% to 21% of patients will experience adverse drug-related events post-discharge, and that the majority of discrepancies in community and hospital medication reconciliation are related to therapeutic duplication (more than one drug from the same class), dose errors, and omitted medication[2,6,60,61].

In summary, effective implementation of medication reconciliation is essential to reduce preventable adverse drug events occurring at the transitions between community and hospital care. More efficient and reliable methods of obtaining the community drug list are critical to improve hospital staff adherence to the medication reconciliation process and reduce unintended discrepancies in community and hospital medication at discharge. Community-based pharmacy records could be used, particularly if the hospital treatment team could automatically retrieve these records. Adherence to medication reconciliation at discharge will likely be improved by providing an automated order entry process that facilitates re-ordering of hospital and community-based medications at discharge. Moreover, the effectiveness of an electronic medication reconciliation module in reducing adverse drug events may be augmented by interventions to improve the successful transmission of treatment discontinuation and change orders to community-based pharmacists and physicians.

Opportunities for Innovative Approaches to Medication Reconciliation in Canada

In Canada, there are unique opportunities to incorporate innovative solutions into medication reconciliation. As a by-product of our public drug insurance program, each province maintains comprehensive records of all dispensed medication for those insured through provincial drug programs. Moreover, almost all provinces provide a comprehensive drug benefit plan for seniors[62], the population that is at greatest risk of adverse drug events[63] and most likely to be hospitalized[64].For over a decade, our group has conducted

research on the potential benefits of providing information on dispensed medication to primary care clinics in Quebec, showing that it reduces inappropriate prescribing[59,59,65-67], and increases access to information to the most vulnerable populations of patients with the lowest education and the most complex drug management[68]. Community pharmacy records are now being made available to emergency departments in Ontario[4], B.C., and Manitoba.
Moreover, with Canada Health Infoway, provinces are advancing the capacity to provide unprecedented drug information access to hospitals, community-based pharmacies and physician offices by implementing provincial pharmanets, which will enable access to information on all active and past prescription drugs for all provincial residents, regardless of their insurance status.

Study Objective

To determine if an electronically-enabled discharge reconciliation intervention that includes:

1) electronic retrieval of community drug lists from community pharmacy records,
2) reconciliation of community and hospital drugs at discharge, and
3) communication of treatment changes to the community-based prescribing physicians &pharmacists

will reduce the risk of adverse drug events (ADEs), ER visits and re-admissions in the 30 days post-discharge compared to usual care.

Pilot Study Results

To determine if the electronic retrieval of the community drug list would add value to the usual care process, we conducted a pilot study at the McGill University Health Center. To access medications from community pharmacies, we used an integrated drug management system (MOXXI) that our research group had already developed to provide on-line access to the Quebec government prescription database of all medications from community pharmacies [54,59,65-74]. The MOXXI system provides near real-time information (within 24 hrs.) on dispensed prescriptions from the 1,800 community pharmacies in Quebec through a secure virtual private network that links to the prescription claims adjudication system of the government insurer (RAMQ). 135 primary care physicians and specialists and 92,500 patients use MOXXI as part of an on-going research program on the impact of information technologies on the quality of care. In the pilot study, we assessed whether the community drug profile identified missing medication at admission, the value for the treatment team, and the number of community providers who would be affected by a discharge reconciliation and communication intervention. In 91 consecutive patients admitted in 2009, we showed that electronically retrieved community pharmacy records identified, on average, 3 additional drugs per patient. For 21% of patients, ≥ 5 drugs were identified (Figure 1).

Figure 1 Differences in the Community Drug Profile
ER Medication History vs. Community Pharmacy Records

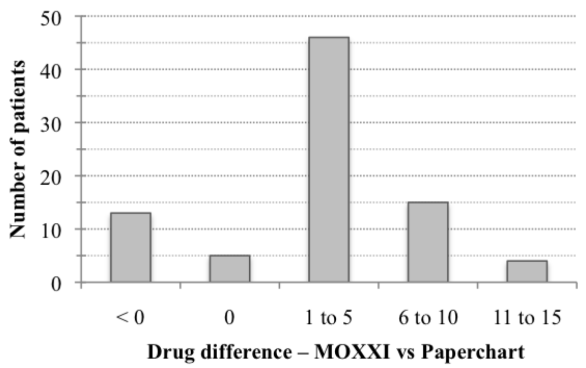

Figure 2 ED Nurses and Physicians (n=33) Perceptions of Value and Access

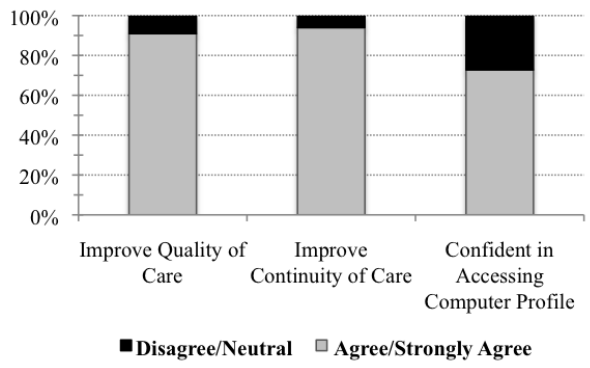

[4] www.health.gov.on.ca/en/public/programs/drugs/access_drug_history/

Over 90% of physicians and nurses who accessed real-time community pharmacy records believed this information improved the quality and continuity of care (Figure 2). Overall, 72.7% were confident in their ability to use a computer to gain access, even though 29% had limited or no prior computer experience. Access to the community drug profile reduced medication history-taking by 2.5 minutes per patient. Moreover, the challenges for staff to access treatment information for traditional medication reconciliation were substantial as 31% of patients had more than one dispensing pharmacy, most had multiple prescribing physicians, and 14.3% had more than 8 (Table 1).

Table 1 The Number of Prescribing Physicians and Dispensing Pharmacies for 91 consecutive patients admitted to the MUHC (April-May, 2008)

Number of Prescribing Physicians	N (%)	Number of Dispensing Pharmacies	N (%)
1 prescribing physician	6 (6.6%)	1 pharmacy	60 (69.0%)
2-4 prescribing physicians	34 (37.4%)	2 pharmacies	18 (20.7%)
5-8 prescribing physicians	38 (41.8%)	≥3 pharmacies	9 (10.4%)
≥9 prescribing physicians	13 (14.3%)		

Study Design

A randomized cluster design will be used to determine if electronically-enabled discharge reconciliation reduces adverse events post discharge. The study population will be patients admitted to medical, surgical, and psychiatric units at the McGill University Health Center. Patients will be stratified by medical, surgical or psychiatric admission, and a cluster randomization approach will be used to allocate the 16 medical, 10 surgical, and 2 psychiatric units to discharge reconciliation and communication versus usual care (Figure 3).

Figure 3 Stratified Random Cluster Design of Electronically-Enabled Discharge Reconciliation vs. Usual Care

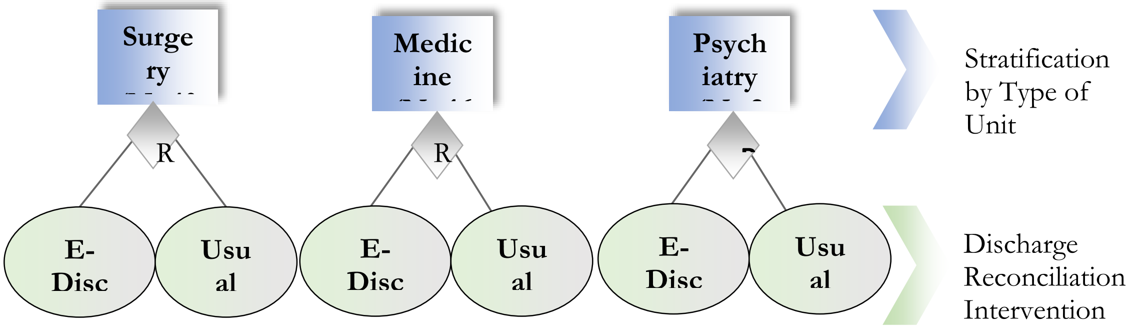

A cluster randomization approach is required to avoid contamination between the two interventions (usual care vs. discharge reconciliation and communication).This is because all medical staff, who are clustered within units, will need to be trained, and provided with on-site support to successfully use computerized order entry to reconcile the community drug list with the in-patient drug list. It will not be possible to randomize patients within a unit without risk of contamination. The primary outcome, adverse drug-related events in 30 days, will be measured by follow-up interview 30 days post-discharge, as this is when post-discharge adverse events occur[2,6], and the secondary outcome, re-admission/ER visit, will be assessed by retrieving complete service utilization claims files from the Quebec insurance agency

Study Population

Eligibility: The target population comprises patients who are at risk of preventable adverse drug events during transitions between community and hospital care. This population represents approximately 60% of the 3.1 million patients admitted to hospital each year in Canada, of whom 93,000 to 428,000 are expected to experience an adverse drug event[75-79]. The study will be conducted at the McGill University Health Centre, a network of teaching hospitals that serve a population of 1.3 million Quebecers from Montreal to James Bay, including a trauma center, two tertiary adult hospitals, a psychiatric facility and a specialized hospital for respiratory disease. Teaching hospitals represent approximately 28% of hospital beds in

Canada[75], but they treat a higher proportion of complex, vulnerable patients where improvements in medication safety would have the greatest benefit. Patients will be eligible for inclusion in the study population if they are: 1) using prescription medication at admission, 2) covered by the Quebec drug insurance plan, 3) admitted from the community, 4) ≥ 18 years old at admission, 5) admitted to a general or intensive care medical, surgical or psychiatric unit, 6) discharged alive. Patients who are cognitively impaired or otherwise unable to provide consent will be included as we have shown that this subpopulation of patients may be at greatest risk of adverse events because of communication problems [80]. Tri-council ethics guidelines deem that this subpopulation should not be excluded from interventions that potentially provide direct benefit to the participant because of the inability to provide informed consent (Article 4.5)[81]. For these patients, the family if present, or the medical director of the admitting unit will authorize study participation. Demographic, clinical and health care service use characteristics of the study population will be retrieved from the admission note, and provincial health care administrative databases in the year before admission and in the two months after discharge to characterize the study population, evaluate the integrity of randomization, assess potential biases related to losses to follow-up, and assess re-admission.
Sample size: We based our sample size requirements on the primary outcome for discharge reconciliation—difference in the rate of adverse drug events—as this binary outcome with correlated patient observations required the maximum sample size (appendix 1). For this outcome, we bracketed expected rates of adverse drug events in the control group based on recent Canadian and American studies [2,9,10] to be between 10% and 19%. We specified an absolute reduction of 5% as being the smallest clinically relevant difference that would be worthwhile to detect. A difference of this magnitude would conservatively result in 96,000 fewer adverse drug events in Canada annually, at an overall estimated annual cost-savings of $240million[1]. Specifying an acceptable Type 1 error of 5%, and Type 2 error of 20%, the estimated sample size for the expected range in possible cluster correlations (r=0.02-0.05) varies from 2,852 to 4,423patient admissions, assuming a baseline rate in the control group of 15%, midway between 10% and 19%.Based on an analysis of re-admission rates in 2008, a cluster correlation no greater than 0.03 is expected, which would mean a sample size of 3,376 is required to detect a 5% reduction in adverse drug events. Based on our prior work, we estimate that 10% of patients will not be reached to complete the post-discharge follow-up interview[9,10], therefore we estimate that we will need to recruit 3,714 patients to achieve our sample size requirements. Overall, in 2008, there were 17,480 admissions to the MUHC, of which 12,236 were admitted to eligible medical, surgical and psychiatric units. Based on our pilot study, approximately 42% (n=5,139) of patients will be eligible for inclusion (i.e. have public drug insurance, ≥ 18 years old, first admission in study period, alive at discharge), and of these 3% will refuse to participate, 45% will not be asked for consent because the staff are too busy, and 52% will consent to participate. By providing staff with support to obtain consent from patients, we expect that we can increase the proportion of eligible patients participating to 60%, or 257 per month. Assuming 10 months per year of active recruitment to account for summer and winter holidays, we estimate that it will take 15 months, or approximately 1.5 years to recruit all patients. With an average length of stay of 8.9 days, we can successfully complete enrollment and follow-up in 18-20 months.

Randomization: The 28 hospital units will be assigned a random number, stratified by type (medicine, surgery, psychiatry), and the default random number generator in SAS will be used to randomly assign units within stratum to electronically enabled discharge reconciliation or usual care. There will be approximately 1,857 patients who are assigned to medication reconciliation at discharge, 1,857 to usual care.

Interventions: Usual Care vs. Electronically Enabled Discharge Medication Reconciliation

Usual Care*:* The community drug list is generally documented at the time of admission. For patients admitted through the emergency department, the triage nurse and ED pharmacist (weekdays only) are responsible for documenting the community medication list in the chart. This may be reviewed and updated by the admitting physician, resident, and/or nurse. When the patient is admitted directly to the unit, the admitting nurse and staff physician/resident are responsible for documenting the medication history. In

addition, there are 14 FTE pharmacists available on weekdays to provide inpatient clinical pharmacy service support for the medical and surgical units, including intensive care. Unit-based pharmacists may provide assistance in obtaining the community drug history, particularly for more complex medication regimens.

At discharge, the attending physician/resident uses the list of current hospital medication, with or without the community drug list (when available), to prescribe the discharge medication. Similar to other Canadian hospitals, the MUHC has implemented an electronic health record (OACIS), that integrates all relevant clinical information from hospital pharmacy, labs, diagnostic imaging, consult reports to be viewed by the treatment team(appendix 2).Active hospital medications can be viewed by accessing the patient's electronic OACIS record, the medication administration chart, or nurse's kardex. The patient is provided with a written discharge prescription to fill at community pharmacy, and may or may not receive verbal or written instructions about new medications or community medications that are being stopped or changed. If the community pharmacist has questions about whether they should continue pre-existing medications that are not included in the discharge medication, they ask the patient, and may call the physician or discharging unit of the hospital.

Electronically enabled Discharge Reconciliation and Communication: The experimental intervention has three components. *First*, at admission, *the community drug list* will be electronically retrieved from the RAMQ using the MOXXI real-time interface, and transferred to the hospital pharmacy system (Figure 4).. In a prior validation study, we have shown that RAMQ prescription claims achieve an accuracy of 100% for the drug dispensed, and 98.5% for the date of dispensing[82]. We will include all drugs where the patient has an active supply of medication in the 7 days prior to admission, as well as provide the treatment team with the option of reviewing all drugs dispensed in the past 6 months using the MOXXI drug profile. The admitting team and hospital pharmacist will verify the list with the patient, add any other medications including over-the-counter

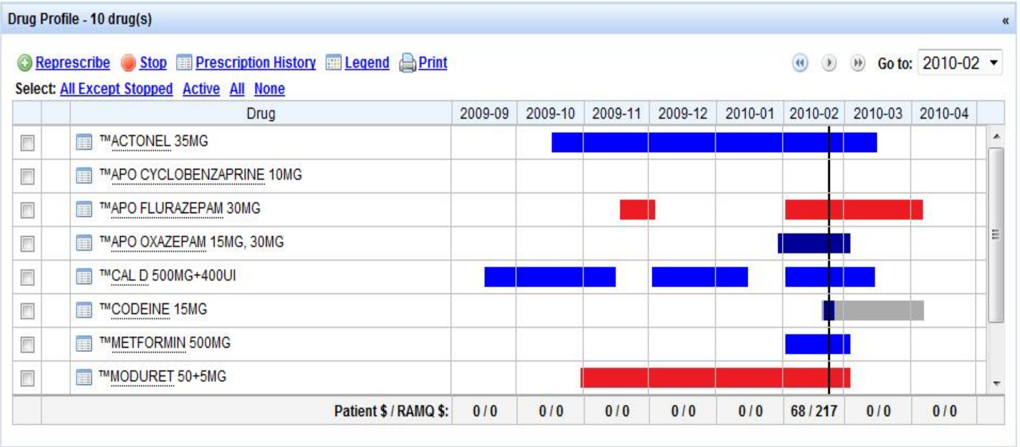

Figure 4 MOXXI Drug Profile: Medication Active Today (Vertical line) and Medication History (6 Months)

and herbal products, and the resulting list will be used to pre-populate the discharge module (Figure 5).

Second, at discharge, the attending physician/resident will write the discharge prescription using the *discharge reconciliation module.*

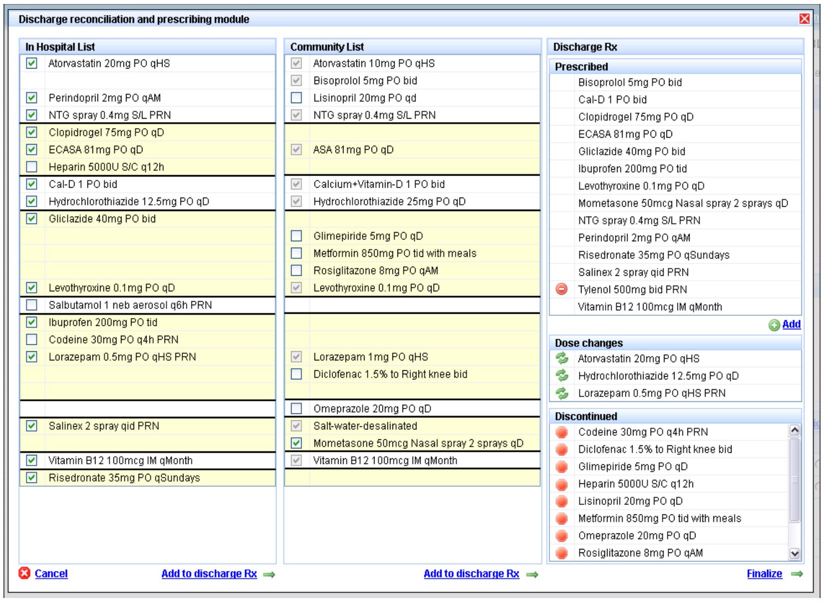

Figure 5 Discharge reconciliation and prescription module

The discharge reconciliation module will be integrated, and directly accessible through the patient's electronic OACIS record. It will display the current active hospital medications, and the verified community-based drug list, sorted by therapeutic class (e.g. antihypertensives, antidepressants) to facilitate reconciliation (Figure 5). The attending physician/resident will "click" on each of the hospital medications that should be included in the discharge prescription, and add any medications from the community drug list that should be continued. All community-based medications that are not included in the discharge prescription will be automatically transferred to the discontinuation section. To assist in viewing the reconciliation process, dose changes will be separated from discontinued medication.

Third, the *discharge communication module* (Figure 6) will facilitate the identification and transfer of information on discontinued and changed medication to the respective dispensing pharmacy (ies) and prescribing physician(s) along with the reasons for these changes. The attending physician/resident will document *the reason for discontinuing or changing the dose of each medication* using a drop-down menu, an approach that has been validated by our group in prior research[72]. A comment field is also available where additional information can be added using free text. The list of prescribed and discontinued medications will be printed, signed by the attending physician/resident, and a copy printed and retained for the chart.

Figure 6 Discharge Communication Module

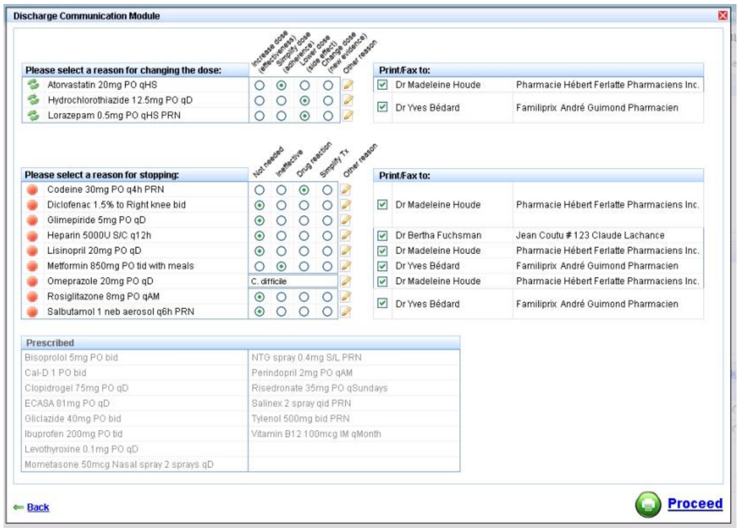

For each discontinued medication or dose change, the dispensing pharmacy and prescribing physician will be identified from the community-based pharmacy claim record. The identity of the prescribing physician and pharmacy must be completed accurately for the pharmacy to receive payment form the RAMQ. Access to this information has been approved for this study by the provincial privacy commission (appendix 3). For each discontinued drug, the name of the patient, drug, and the reason for discontinuation, attending physician, hospital, and discharge date will be faxed to the respective dispensing pharmacy and prescribing physician. Fax numbers for prescribing physicians will be retrieved from the Collège des médecins du Québec who will provide the research team with a monthly updated file of all licensed physicians in Quebec, their telephone and fax numbers. Fax numbers for community pharmacies will be retrieved from Vigilance, a pharmacy software provider that receives a monthly update of this information from the Association Québécoise des Pharmaciens Propriétaires, that is used by pharmacies to fax transferred prescriptions from one pharmacy to another. To

minimize the risk of transmitting confidential information to incorrect fax numbers, we will call the pharmacy and physician's office before faxing patient information to verify the fax number.

Adherence to the Intervention: Our main challenge will be to ensure that attending physicians/residents have sufficient training, motivation, and support to use the medication reconciliation module for the discharge prescription. The four conditions needed for successful adoption will be incorporated into the implementation of discharge reconciliation[83]. First, we will train local leadership within each unit to champion the discharge reconciliation process comprising the unit service chief, head nurse, and liaison pharmacist. Second, we will pre-test and calibrate the user interface to minimize workflow disruption and maximize efficiency gains. Based on our experience of instituting electronic prescribing in the primary care setting, we expect that we can save 3 or more minutes per discharge prescription, by allowing relevant hospital and community-based prescriptions to be copied to the discharge prescription[67]. Third, we will prepare, with the clinical champions, a blitz launch in each unit that will feature on-site support for completing the discharge prescription in each unit for the first 2 weeks. We have successfully used nursing and medical students, who are typically adept computer users, to provide real-time coaching and support for using new clinical computer applications. Last, we will monitor adoption by using application audit trails, and use this information to identify and remedy problems that may exist on certain units.

Bias Control The main challenge in *bias control* is the inability to blind staff to treatment assignment, and potential co-intervention. To control bias, the research assistant, expert panel, and analyst assessing the outcome of treatment will be blinded to unit and treatment allocation status. To assess bias related to possible co-interventions, the project coordinator will conduct a monthly review with the unit directors and hospital pharmacy to assess co-interventions (new initiatives that may modify the study outcomes), and we will use sensitivity analysis to assess the potential impact on the study outcomes. As the involvement of the hospital pharmacist in the patient's care likely reduces the risk of adverse events (e.g. through medication review), we will retrieve this information from the chart and assess whether hospital pharmacist intervention (yes vs. no) is a confounder by including this information as a patient-level variable in the analysis. In addition, as cluster randomization may not produce patients groups who have an equivalent risk of adverse events post-discharge (i.e. because randomization is by unit rather than by patient), we will assess whether patients admitted to the usual care vs. e-discharge units had a similar rate of hospitalizations, and ER visits in the 12 months prior to admission using RAMQ medical service data retrieved for each patient, and include prior ER/hospitalization history in the analysis to determine if it confounds the estimated effect of the intervention.

Outcome Assessment

Adverse Drug Events: Adverse drug events are defined as the occurrence of new signs and symptoms post-discharge that are judged to have a moderate to high probability of being drug-related. Adverse drug events will be assessed by: a) collecting self-reported patient information 30 days post-discharge, b) retrieving chart and administrative data on drugs that were started, stopped or continued at discharge as well as acute and chronic health problems, and c) reviewing and adjudicating the presence of an adverse event and the probability of it being drug related by a blinded expert panel review of each patient's chart and post-discharge interview data using the Naranjo criteria.

1) Self-Report Interview: Patient self-report will be used because it is the most sensitive method of adverse drug event detection, identifying an additional 28% of adverse events compared to medical chart review, of which 13% are considered serious[84]. A modification of the Australian two-step adverse reaction and drug event report will be employed to solicit patient feedback on potential adverse events, and their characteristics[85]. Both questionnaire and telephone interview versions of this protocol have been assessed. As the telephone version provides higher sensitivity of event detection, we have elected to use the latter[85]. In the first step, patients will be asked to report any new health problem or change in their condition since discharge that they think may be related to their medication(s). In the second step, a review of systems is

conducted using directed probes for changes in systems-related symptoms or signs that may be drug-related (e.g. skin rash, cough). For positive responses, patients will be asked to describe each new problem, and indicate when it started in relation to the initiation of drug treatment post-discharge.

Most adverse events occur early in the post-discharge period, therefore, we will conduct the follow-up interview in the first 25 to 30 days post-discharge[2,6]. A trained research assistant will conduct patient follow-up interviews, **blinded** to intervention status. Five attempts will be made to contact the patient. If a patient cannot be reached successfully, we will send a questionnaire version of the adverse reaction and event report by mail, with an accompanying letter from their attending physician, and self-addressed stamped envelope for return. We will assess the quality of the interview and documentation provided by the research assistant by a monthly quality control check using standardized patient interviews. These quality control methods, that have been employed by our team in past research, will allow us to estimate the agreement between actual documentation and expected documentation, where the standardized case represents the gold standard[86,87]. Additional training will be employed as needed to correct deviations from the protocol. Patient interview data will be entered into an ACCESS database.

Chart Abstraction: For each patient, a trained nurse abstractor, blinded to intervention status, will abstract the medical chart. We will abstract dates of admission and discharge, admitting and discharge unit, patient demographics, health problems at admission and discharge, major procedures (surgeries, treatment interventions), and medications at admission, during the hospital stay and at discharge, including medications that were stopped and changed by the in-hospital treatment team. Health problems will be coded using ICD10, medications by generic chemical code using Anatomic-Therapeutic Classification system, and procedures by Canadian Classification and Diagnostic and Therapeutic Procedures (CCP). Data will be entered using the MOXXI chart abstraction system as it permits text entries (e.g. hydrochlorithiazide 10mg, rheumatoid arthritis) to be mapped to the respective classification system and stored by patient identifier in the MOXXI Oracle database. A case summary record, that includes the community drug list, hospital drug list, discharge abstract, discharge medications and patient self-report, will be prepared for causality assessment by the expert panel.

Expert Review and ADE Assessment: A number of methods have been developed for expert panels to assess probability that a given event is drug-related on the basis of patient data. None of the scoring systems are accepted as a gold standard, but the Naranjo criteria for ADE assessment is the most widely used method of ADE causality assessment in practice[88]. The Naranjo instrument uses patient self-report or chart review data to assess the presence or absence of ten criteria related to the adverse event (e.g. renal failure). The relative importance of each criterion is weighted from -1 to +2. The sum of criteria-specific weights is used to classify the probability that the event was drug-related into one of 4 categories: definite, probable, possible or doubtful[89]. Inter-rater agreement in assessing events using Naranjo criteria is good to excellent (kappa: 0.69-0.86) and intra-rater agreement varies between kappa=0.64-0.95. Naranjo scored events correlate with other similar measures such as the Kramer ADE probability assessment instrument (r = 0.82)[90]. Gold standard ADEs will be defined as those with a causality assessment score classified as definite or probable. Three clinicians who are part of the study team will assess the Naranjo criteria. Each clinician will receive the patient self-report for all patients with a positive response to the open-ended query of about adverse effects, or system-specific probes, as well as all clinical information retrieved from the chart. Clinicians will be **blinded** to the patient's intervention status (usual care vs. discharge reconciliation). Each clinician will independently score each patient on the 10 Naranjocriteria[89]. Each clinician's score will be used to classify that patient as having a definite (score≥9), probable (score 5-8), possible (score 1-4) or doubtful (score=0) adverse drug event. All patient cases where there is a difference in final classification will be reviewed and discussed by the panel of three clinicians to reach a consensus classification. We will test inter-rater agreement using 10 training charts before the start of the study, and each month during follow-up using one standardized chart per month. Agreement in scoring on the training and monthly quality control charts will be assessed by an intra-class correlation and in the Naranjo classification by weighted kappa.

ER Visit/Hospital re-admission: All visits to the emergency room and/or hospital re-admission in the 30 days post-discharge will be measured using the RAMQ provincial health care databases. This approach ensures that all ER visits and re-admissions are included, not just those occurring to at the MUHC. This is

important because ambulances will transport individuals to the closest, open emergency room/hospital which often is not the discharging institution. Almost all hospital-based physicians in Quebec are remunerated on a fee-for-service basis[91], and for each medical service delivered, physicians are required to accurately record the treating establishment, and the location of the service (e.g. ICU, ER, day hospital, inpatient unit), because location and type of establishment determine the level of remuneration. For each consenting patient, the RAMQ will retrieve all records of services provided in the month after discharge. Patients will be classified as having an ER visit if they have a record of service with a location of a hospital emergency room, and a re-admission if they have a service delivered from an in-patient general hospital unit. In secondary analysis, we will retrieve all ICD10 diagnostic codes recorded for ER visits and re-admissions to provide descriptive information on potential reasons for the visit/re-admission.

Data Management and Analysis

Four sources of data will be assembled and linked to address the study objective: 1) abstracted medical chart documentation of patient demographics, admission and discharge dates, the community drug list, discharge prescription, and admitting and discharge diagnoses, 2) adverse events information and assessment post-discharge, 3) the RAMQ medical services and prescription claims data, and 4) co-intervention data collected by the study co-ordinator. All data will be managed in an Oracle database, and files for individual patients will be linkable through a study identification number and Quebec medicare number, with nominal information retained in a separate encrypted file.

The integrity of randomization will be assessed by characterizing the age, sex, baseline number of visits, hospitalizations, medications, and co-morbidity (using the Charlson co-morbidity index[92], a weighted index of conditions that increase the risk of mortality) of patients randomized to discharge reconciliation and usual care. The CONSORT guidelines will be followed to document the eligibility and follow-up of patients and inpatient units (clusters) in the trial[93]. The provincial health administrative data will be used to characterize bias related to patients lost to follow-up after discharge for whom adverse drug events cannot be measured, based on the secondary outcome (re-admission/ER visits) as this can be measured on all patients.

An intention to treat approach will be used to analyze study results. To determine whether discharge reconciliation reduces the risk of adverse drug events post-discharge, we will use logistic regression within a generalized estimating equation framework to account for clustering of patients within unit[94]. The presence of a probable or definite adverse event (yes vs. no) will be the outcome variable, hospital unit will be the defined clustering factor, and an exchangeable correlation matrix will be used to account for clustering of patients within unit. Discharge reconciliation will be fit as a dummy variable, using usual care as the reference group. We will use the same approach to assess the secondary outcome, ER visits and/or hospital re-admission. For both the primary and secondary outcome, we will assess, whether adjustment for co-interventions and baseline differences between patients in the usual care and intervention arm confound the effect of the intervention. In secondary analysis, we will assess whether the effect of the intervention is modified by hospital unit type (medicine vs. surgery vs. psychiatric), and patient characteristics that are associated with a higher risk of adverse events (age, number of medications at discharge) by including respective interaction terms in the logistic model and testing their significance using the Wald chi-square statistic.

Monitoring Study Outcomes: Trial Termination Decision Rules and Other Ethical Considerations

To monitor any unintended adverse effects arising from the study intervention(s), we will establish an independent data monitoring board, chaired by Dr David Bates (Harvard University), and including Dr Gordie Schiff (Harvard University), and Dr Anne Holbrook (McMaster University), scientists who provide pre-eminent expertise in adverse drug events, computerization of health care, and biostatistics. The data monitoring board will monitor quarterly statistics on hospital re-admission and ER visit rates to assess unintended effects, blinded to study group. The study team will be responsible for providing any additional statistical information that are deemed necessary by the data monitoring board to ensure patient safety.

Patients who may not be able to consent at the time of admission because they are cognitively impaired or unable to communicate will be followed up by the research assistant to determine if the patient is able to provide an informed consent. Patients who are able to consent and choose not to enroll at that point will be excluded from the study.

The Team

Dr Tamblyn, James McGill Chair and Professor in the Departments of Medicine, and Epidemiology & Biostatistics will assume overall leadership for trial conduct and analysis. Dr Buckeridge, the Canada Research Chair in Public Health Informatics, will take the lead in the development and integration of the discharge reconciliation and communication modules. Dr Forster will assume leadership for adverse drug event assessment, using methods of adverse drug event detection he has developed and tested with studies conducted in Boston, Ottawa and most recently Montreal. Drs Huang, Rochefort, Winslade, and Meguerditchian will assume responsibility for clinical leadership in implementation of the study protocol in the medical, surgical, and psychiatric units with Huang taking leadership for medicine, Meguerditchian for surgery, Winslade for pharmacy and Rochefort for nursing. The clinical leads will also assume responsibility for the blinded assessment of adverse drug events, coordinated by Dr Forster.

Expected Contribution & Knowledge Translation

Medication reconciliation at discharge from hospital is expected to reduce unintended discrepancies in community and hospital-based treatment, and preventable adverse drug-related events. We expect that the intervention that will be evaluated in this trial may improve adherence to medication reconciliation at discharge, the accuracy of the community drug history, and effective communication of hospital-based treatment changes to community-based care providers. The solutions we will test are available in every Canadian province and can be amplified to include all patients admitted when provincial pharmanets are fully implemented and made accessible to hospital and community-based care teams. The results of this study will inform future directions. If we find that the intervention reduces adverse drug events, it will support policy-directed quality investments in computerizing and training hospital staff with the capacity to use pharmacy-based records and a discharge reconciliation module to improve medication reconciliation. It will also generate the key requirements for medication reconciliation that can be applied by Accreditation Canada in future hospital accreditation standards, as well specify the functional requirements for software vendors.

Our knowledge translation strategy will target three groups. The first group will be those responsible for establishing standards, requirements and priorities for health IT investment: Canada Health Infoway and provincial and regional CIOs. We will request an invitation to present to the Infoway board of directors (who represent these stakeholders), present at their annual meeting (COACH), and transmit a 1-page summary of key messages to this network via mail/e-mail. Second, we will collaborate with Accreditation Canada and the Canadian Healthcare Organization to develop a communication package that will be useful for its hospital member organizations to exchange essential information from this study for their respective hospital members. We anticipate that we may partner with both Canada Health Infoway and Accreditation Canada in putting together a set of regional workshops to provide more in-depth transfer of expertise. Third, we will communicate our findings to our academic and research colleagues through conference presentations and submission of manuscripts for publication[95].

References

(1) Bates DW Spell N, Cullen DJ et al. The costs of adverse drug events in hospitalized patients. Adverse Drug Events Prevention Study Group. *JAMA.* 1997;277:307-311.

(2) Leape LL, Bates DW, Cullen DJ et al. Systems analysis of adverse drug events. ADE Prevention Study Group. *JAMA.* 1995;274:35-43.

(3) LaPointe NM, Jollis JG. Medication errors in hospitalized cardiovascular patients. *Arch Intern Med.* 2003;163:1461-1466.

(4) Cornish PL, Knowles SR, Marchesano R et al. Unintended medication discrepancies at the time of hospital admission. *Arch Intern Med.* 2005;165:424-429.

(5) Tam VC, Knowles SR, Cornish PL, Fine N, Marchesano R, Etchells EE. Frequency, type and clinical importance of medication history errors at admission to hospital: a systematic review. *CMAJ.* 2005;173:510-515.

(6) Pippins JR, Gandhi TK, Hamann C et al. Classifying and predicting errors of inpatient medication reconciliation. *J Gen Intern Med.* 2008;23:1414-1422.

(7) Akwagyriam I, Goodyer LI, Harding L, Khakoo S, Millington H. Drug history taking and the identification of drug related problems in an accident and emergency department. *J Accid Emerg Med.* 1996;13:166-168.

(8) Lau HS, Florax C, Porsius AJ, De BA. The completeness of medication histories in hospital medical records of patients admitted to general internal medicine wards. *Br J Clin Pharmacol.* 2000;49:597-603.

(9) Forster AJ, Clark HD, Menard A et al. Adverse events among medical patients after discharge from hospital. *CMAJ.* 2004;170:345-349.

(10) Forster AJ, Murff HJ, Peterson JF, Gandhi TK, Bates DW. The incidence and severity of adverse events affecting patients after discharge from the hospital. *Ann Intern Med.* 2003;138:161-167.

(11) Coleman EA, Smith JD, Raha D, Min SJ. Posthospital medication discrepancies: prevalence and contributing factors. *Arch Intern Med.* 2005;165:1842-1847.

(12) Paparella S. Medication reconciliation: doing what's right for safe patient care. *J Emerg Nurs.* 2006;32:516-520.

(13) Using medication reconciliation to prevent errors. *Jt Comm J Qual Patient Saf.* 2006;32:230-232.

(14) Saufl NM. Reconciliation of medications. *J Perianesth Nurs.* 2006;21:126-127.

(15) Manno MS, Hayes DD. Best-practice interventions: how medication reconciliation saves lives. *Nursing.* 2006;36:63-64.

(16) Vira T, Colquhoun M, Etchells E. Reconcilable differences: correcting medication errors at hospital admission and discharge. *Qual Saf Health Care.* 2006;15:122-126.

(17) Barker KN, Flynn EA, Pepper GA, Bates DW, Mikeal RL. Medication errors observed in 36 health care facilities. *Arch Intern Med.* 2002;162:1897-1903.

(18) Kemp LO, Narula P, McPherson ML, Zuckerman I. Medication Reconciliation in Hospice : A Pilot Study. *Am J Hosp Palliat Care.* 2009;26:193-199.

(19) Ledger S, Choma G. Medication reconciliation in hemodialysis patients. *CANNT J.* 2008;18:41-43.

(20) Gleason KM, Groszek JM, Sullivan C, Rooney D, Barnard C, Noskin GA. Reconciliation of discrepancies in medication histories and admission orders of newly hospitalized patients. *Am J Health Syst Pharm.* 2004;61:1689-1695.

(21) Vigoda MM, Gencorelli FJ, Lubarsky DA. Discrepancies in medication entries between anesthetic and pharmacy records using electronic databases. *Anesth Analg.* 2007;105:1061-5, table.

(22) Weingart SN, Cleary A, Seger A et al. Medication reconciliation in ambulatory oncology. *Jt Comm J Qual Patient Saf.* 2007;33:750-757.

(23) Groeschen H. Electronic system improves medication reconciliation rates. *American Journal Health-Syst Pharm.* 2007;64:1894.

(24) What's expected for med reconciliation? *OR Manager.* 2008;24:21, 23.

(25) Rozich JD, Howard RJ, Justeson JM, Macken PD, Lindsay ME, Resar RK. Standardization as a mechanism to improve safety in health care. *Jt Comm J Qual Saf.* 2004;30:5-14.

(26) Bridgeman, Patrick J. and Rynn, Kevin O. Medication reconciliation in the emergency department. American Journal of Health-System Pharmacy 65[24], 2325-2326. 12-15-2008. American Society of Health System Pharmacists.

(27) Meds reconciliation summit promises more clarification. *ED Manag.* 2008;20:suppl-4.

(28) Agrawal A, Wu WY. Reducing medication errors and improving systems reliability using an electronic medication reconciliation system. *Jt Comm J Qual Patient Saf.* 2009;35:106-114.

(29) Lesselroth BJ, Felder RS, Adams SM et al. Design and implementation of a medication reconciliation kiosk: the Automated Patient History Intake Device (APHID). *J Am Med Inform Assoc.* 2009.

(30) Poon EG, Blumenfeld B, Hamann C et al. Design and Implementation of an Application and Associated Services to Support Interdisciplinary Medication Reconciliation Efforts at an Integrated Healthcare Delivery Network. *J Am Med Inform Assoc.* 2006;13:581-592.

(31) Turchin A, Hamann C, Schnipper JL et al. Evaluation of an inpatient computerized medication reconciliation system. *J Am Med Inform Assoc.* 2008;15:449-452.

(32) Clay BJ, Halasyamani L, Stucky ER, Greenwald JL, Williams MV. Results of a medication reconciliation survey from the 2006 Society of Hospital Medicine national meeting. *J Hosp Med.* 2008;3:465-472.

(33) Mersfelder TL, Bickel RJ. Inpatient medication history verification by pharmacy students. *Am J Health Syst Pharm.* 2008;65:2273-2275.

(34) Miller SL, Miller S, Balon J, Helling TS. Medication reconciliation in a rural trauma population. *Ann Emerg Med.* 2008;52:483-491.

(35) van den Bemt PM, van den BS, van Nunen AK, Harbers JB, Lenderink AW. Medication reconciliation performed by pharmacy technicians at the time of preoperative screening. *Ann Pharmacother.* 2009;43:868-874.

(36) Lubowski TJ, Cronin LM, Pavelka RW, Briscoe-Dwyer LA, Briceland LL, Hamilton RA. Effectiveness of a medication reconciliation project conducted by PharmD students. *Am J Pharm Educ.* 2007;71:94.

(37) Schwarz M, Wyskiel R. Medication reconciliation: developing and implementing a program. *Crit Care Nurs Clin North Am.* 2006;18:503-507.

(38) Haig K. Medication reconciliation. *Am J Med Qual.* 2006;21:299-303.

(39) Bayley KB, Savitz LA, Maddalone T, Stoner SE, Hunt JS, Wells R. Evaluation of patient care interventions and recommendations by a transitional care pharmacist. *Therapeutics and Clinical Risk Management.* 2007;3:695-703.

(40) Varkey P, Resar RK. Medication reconciliation implementation in an academic center. *Am J Med Qual.* 2006;21:293-295.

(41) Kramer JS, Hopkins PJ, Rosendale JC et al. Implementation of an electronic system for medication reconciliation. *Am J Health Syst Pharm.* 2007;64:404-422.

(42) Isaac LM, Tamblyn RM, and the McGill Calgary Drug Research Team. Compliance and Cognitive Function: A Methodological Approach to Measuring Unintentional Errors in Medication Compliance in the Elderly. *The Gerontologist.* 1993;33 (6):772-781.
(43) Karnon J, Campbell F, Czoski-Murray C. Model-based cost-effectiveness analysis of interventions aimed at preventing medication error at hospital admission (medicines reconciliation). *J Eval Clin Pract.* 2009;15:299-306.
(44) Orrico KB. Sources and types of discrepancies between electronic medical records and actual outpatient medication use. *J Manag Care Pharm.* 2008;14:626-631.
(45) Schoen C, Osborn R, Huynh PT, Doty M, Peugh J, Zapert K. On the front lines of care: primary care doctors' office systems, experiences, and views in seven countries. *Health Aff (Millwood).* 2006;25:w555-w571.
(46) Fulford MD, Berger BA, Newton DS. The Relationship between Computerization and Profitability in Independent Community Pharmacies. *Journal of Pharmaceutical Marketing & Management.* 1988;3:75-98.
(47) Sikka R, Xia F, Aubert RE. Estimating medication persistency using administrative claims data. *Am J Manag Care.* 2005;11:449-457.
(48) Steiner JF, Prochazka AV. The assessment of refill compliance using pharmacy records: methods, validity, and applications. *J Clin Epidemiol.* 1997;50:105-116.
(49) Choo PW, Rand CS, Inui TS et al. Validation of patient reports, automated pharmacy records, and pill counts with electronic monitoring of adherence to antihypertensive therapy. *Med Care.* 1999;37:846-857.
(50) Bails D, Clayton K, Roy K, Cantor MN. Implementing online medication reconciliation at a large academic medical center. *Jt Comm J Qual Patient Saf.* 2008;34:499-508.
(51) Schenkel S. The unexpected challenges of accurate medication reconciliation. *Ann Emerg Med.* 2008;52:493-495.
(52) Aarts J, Koppel R. Implementation of Computerized Physician Order Entry in Seven Countries. *Health Aff.* 2009;March/April:404-414.
(53) Weingart SN, Toth M, Sands DZ, Aronson MD, Davis RB, Phillips RS. Physicians' decisions to override computerized drug alerts in primary care. *Arch Intern Med.* 2003;163:2625-2631.
(54) Tamblyn R, Huang A, Taylor L et al. A randomized trial of the effectiveness of on-demand versus computer-triggered drug decision support in primary care. *J Am Med Inform Assoc.* 2008;15:430-438.
(55) Beers MH, Dang J, Hasegawa J, Tamai IY. Influence of hospitalization on drug therapy in the elderly. *J Am Geriatr Soc.* 1989;37:679-683.
(56) Jackevicius CA, Paterson JM, Naglie G. Concordance between discharge prescriptions and insurance claims in post-myocardial infarction patients. *Pharmacoepidemiol Drug Saf.* 2007;16:207-215.
(57) Jack BW, Chetty VK, Anthony D et al. A reengineered hospital discharge program to decrease rehospitalization. *Ann Intern Med.* 2009;150:178-187.
(58) Tamblyn, R. M., McLeod, P., Abrahamowicz, M., Berkson, L., Dauphinee, D., Gayton, D., Grad, R., Huang, A., Isaac, L., Monette, J., Sampalis, J., Schnarch, B., and Snell, L. Multi-Physician Involvement and Questionable High Risk Prescribing in the Elderly. The Canadian Pharmacoepidemiology Forum, Toronto . 1993.
(59) Tamblyn RM. Improving patient safety through computerized drug management: The devil is in the details. *HealthCare Papers.* 2004;5:52-68.

(60) Kanjanarat P, Winterstein AG, Johns TE, Hatton RC, Gonzalez-Rothi R, Segal R. Nature of preventable adverse drug events in hospitals: a literature review. *Am J Health Syst Pharm.* 2003;60:1750-1759.

(61) Agrawal A, Aronson JK, Britten N et al. Medication errors: problems and recommendations from a consensus meeting. *Br J Clin Pharmacol.* 2009;67:592-598.

(62) National Pharmaceuticals Strategy (NPS). Federal Government of Canada . 2006.

(63) Gurwitz JH, Avorn J. Old age - Is it a risk for adverse drug reactions? *Agents & Actions - Supplements.* 1990;29:13-25.

(64) 2006 Canadian Hypertension Education Program Recommendations. Canadian Medical Association. 2006. CMA Infobase, Clinical Practice Guidelines.

(65) Tamblyn R, Huang A, Perreault R et al. The medical office of the 21st century (MOXXI): effectiveness of computerized decision-making support in reducing inappropriate prescribing in primary care. *CMAJ.* 2003;169:549-556.

(66) Taylor LK, Kawasumi Y, Bartlett G, Tamblyn R. Inappropriate prescribing practices: the challenge and opportunity for patient safety. *Healthc Q.* 2005;8 Spec No:81-85.

(67) Tamblyn R, Huang A, Kawasumi Y et al. The Development and Evaluation of an Integrated Electronic Prescribing and Drug Managment System for Primary Care. *J Am Med Inform Assoc.* 2006;13:148-159.

(68) Kawasumi Y, Tamblyn R, Platt R, Ernst P, Abrahamowicz M, Taylor L. Evaluation of the use of an integrated drug information system by primary care physicians for vulnerable population. *International Journal of Medical Informatics.* 2008;In Press, Corrected Proof.

(69) Poissant L, Pereira J, Tamblyn R, Kawasumi Y. The impact of electronic health records on time efficiency of physicians and nurses: a systematic review. *J Am Med Inform Assoc.* 2005;12:505-516.

(70) Bartlett G, Tamblyn R, Taylor L, Poissant L, Kawasumi Y. Non-participation bias in health services research using data from an integrated electronic prescribing project: the role of informed consent. *Acta Bioethica.* 2005;11:145-159.

(71) Motulsky A, Winslade N, Tamblyn R, Sicotte C. The impact of electronic prescribing on the professionalization of community pharmacists: a qualitative study of pharmacists' perception. *J Pharm Pharm Sci.* 2008;11:131-146.

(72) Eguale T, Tamblyn R, Winslade N, Buckeridge D. Detection of adverse drug events and other treatment outcomes using an electronic prescribing system. *Drug Saf.* 2008;31:1005-1016.

(73) Reidel K, Tamblyn R, Patel V, Huang A. Pilot Study of an Interactive Voice Response System to Improve Medication Refill Compliance. *BMC Med Inform Decis Mak.* 2008;8:46.

(74) Tamblyn R, Reidel K, Huang A et al. Increasing the Detection and Response to Adherence Problems with Cardiovascular Medication in Primary Care through Computerized Drug Management Systems. *Med Decis Making.* 2009;IN PRESS.

(75) Hospital Trends in Canada - Results of a Project to Creata a Historical Series of Statistical and financial Data for Canadian Hospitals Over Twenty-Seven Years. 1-85. 2005. Ottawa, Ontario, Canadian Institute for Health Information.

(76) Grymonpre RE, Mitenko PA, Sitar DS, Aoki FY, Montgomery PR. Drug-Associated Hospital Admissions in Older Medical Patients. *J Am Geriatr Soc.* 1988;36:1092-1098.

(77) Ives TJ, Bentz EJ, Gwyther RE. Drug-related admissions to a family medicine inpatient service. *Arch Intern Med.* 1987;147:1117-1120.

(78) Hurwitz N. Predisposing Factors in Adverse Reactions to Drugs. *Br Med J.* 1969;1:536-539.

(79) May FE, Stewart RB, Cluff LE. Drug Interactions and Multiple Drug Administration. *Clin Pharmocol Therap.* 1977;22:322.
(80) Bartlett G, Blais R, Tamblyn R, Clermont RJ, MacGibbon B. Impact of patient communication problems on the risk of preventable adverse events in acute care settings. *CMAJ.* 2008;178:1555-1562.
(81) Interagency Advisory Panel on Research Ethics. Tri-Council Policy Statement: Ethical Conduct for Research Involving Humans - Draft 2nd Edition. 1-154. 2008. Ottawa, Interagency Secretariat on Research Ethics.

(82) Tamblyn RM, Lavoie G, Petrella L, Monette J. The Use of Prescription Claims Databases in Pharmacoepidemiological Research: The Accuracy and Comprehensiveness of the Prescription Claims Database in Quebec. *J Clin Epidemiol.* 1995;48(8):999-1009.
(83) Bates DW, Kuperman GJ, Wang S et al. Ten commandments for effective clinical decision support: making the practice of evidence-based medicine a reality. *J Am Med Inform Assoc.* 2003;10:523-530.
(84) Gandhi TK, Weingart SN, Borus J et al. Adverse drug events in ambulatory care. *N Engl J Med.* 2003;348:1556-1564.
(85) Mitchell AS, Henry DA, Hennrikus D, O'Connel DL. Adverse drug reactions: Can consumers provide early warning? *Pharmacoepidemiol Drug Saf.* 1994;3:257-264.
(86) Tamblyn RM, Abrahamowicz M, Schnarch B, Colliver J, Benaroya S, Snell L. Can Standardized Patients Predict Real-Patient Satisfaction with the Doctor-Patient Relationship? *Teach Learn Med.* 1994;6 (1):36-44.
(87) Tamblyn R, Berkson L, Dauphinee WD et al. Unnecessary prescribing of NSAIDs and the management of NSAID-related gastropathy in medical practice. *Ann Intern Med.* 1997;127:429-438.
(88) Kelly WN. The quality of published adverse drug event reports. *Ann Pharmacother.* 2003;37:1774-1778.
(89) Naranjo CA, Busto U, Sellers EM et al. A method for estimating the probability of adverse drug reactions. *Clin Pharmacol Ther.* 1981;30:239-245.
(90) Ovens H, Talbot Y, Harris F, Newman B. Hypnosis training enhances communication skills. *Medical Teacher.* 1990;12(3/4):357-361.
(91) Regie de l'assurance-maladie du Quebec. Statistiques Annuelles. ISBN 2 -550 -30856-5, 46-48. 2000. Quebec, Quebec, Regie de l'assurance-maladie du Quebec.
(92) Charlson ME, Pompei P, Ales KL, MacKenzie CR. A New Method of Classifying Prognostic Comorbidity in Longitudinal Studies: Development and Validation. *Journal of Chronic Disease.* 1987;40(5):373-383.
(93) Campbell MK, Elbourne DR, Altman DG. CONSORT statement: extension to cluster randomised trials. *Br Med J.* 2004;328:702-708.
(94) Zeger SL, Liang KY, Albert PS. Models for longitudinal data: a generalized estimating equation approach. *Biometrics.* 1988;44:1049-1060.
(95) Starren J, Hripcsak G, Sengupta S et al. Columbia University's Informatics for Diabetes Education and Telemedicine (IDEATel) project: technical implementation. [see comments.]. *Journal of the American Medical Informatics Association.* 2002;9:25-36.
