# Supplementary material for: Effect of an Electronic Medication Reconciliation Intervention on Adverse Drug Events: A Cluster Randomized Trial
Source: JAMA Netw Open. 2019 Sep 20;2(9):e1910756. doi: 10.1001/jamanetworkopen.2019.10756 (PMC6755531; doi:10.1001/jamanetworkopen.2019.10756)
Supplement: Supplement 2. — eAppendix. Analysis eFigure 1. Screenshot of the RightRx User Interface at Discharge eFigure 2. Discharge Prescription eFigure 3. ADE Adjudication Forms eReferences. [file jamanetwopen-2-e1910756-s002.pdf]

Supplementary Online Content

Tamblyn R, Abrahamowicz M, Buckeridge DL, et al. Effect of an electronic medication reconciliation intervention on adverse drug events: a cluster randomized trial. *JAMA Netw Open*. 2019;2(9):e1910756. doi:10.1001/jamanetworkopen.2019.10756

eAppendix. Analysis
eFigure 1. Screenshot of the RightRx User Interface at Discharge
eFigure 2. Discharge Prescription
eFigure 3. ADE Adjudication Forms
eReferences.

This supplementary material has been provided by the authors to give readers additional information about their work.

© 2019 Tamblyn R et al. *JAMA Network Open.*

eAppendix. Analysis

To account for clustering, and avoid the inflation of the Type I error that occurs with a small number of clusters[1-3], 95% confidence intervals for the adjusted intervention effect of the were estimated using the non-parametric two-step cluster bootstrap method, based on 10,000 bootstrap samples. The two-step cluster bootstrap approach was previously developed and validated in extensive simulations for complex analyses of clustered data[4]. The advantage of this method is that it avoids both: (i) arbitrary assumptions about the covariance structure of the residuals that may affect the results of conventional parametric clustering methods such as GEE or generalized mixed effects models, and (ii) reliance on the asymptotic large-sample theory.

2-step cluster bootstrap samples were generated in two steps [4]. In the first step, within each trial arm, the two clusters (hospital units) originally randomized to this arm were resampled 10,000 times, with replacement. For each of the 10,000 (j=1,.., 10,000) first step cluster solutions, in the second step, the N(i) individual patients within each cluster "i" selected in the first step were resampled N(i) times with replacement, to create a bootstrap resample representation of a given unit, i.e. a random bootstrap 'mutation' of the original cluster-specific sample. The final j- th (j=1,.., 10,000) 2-step bootstrap resample was then created by combining the results of the 2nd step resampling for each of the four clusters (hospital units) resampled at the 1st step to generate the bootstrap resamples. For each of the resulting 10,000 bootstrap resamples generated, we then used the same multivariable logistic regression model as in the original analyses to estimate the adjusted treatment effect for this resample. Then, the 2.5th and 97.5th percentiles of the resulting distribution of the OR's for the intervention effect, across the 10,000 bootstrap resamples, were used to estimate the 2-step bootstrap-based 95% confidence interval (CI) of the treatment effect.

It should be noted that previous simulations indicated that the 95% CI's yielded by the above 2-step bootstrap approach are somewhat conservative, i.e. too wide, resulting in higher than nominal (95%) coverage [4]. In fact, to accurately account for clustering, it will be typically sufficient to perform only the 1st step, i.e. bootstrap only the clusters, without bootstrapping individual subjects within the clusters [4]. However, in our specific context, with only 2 clusters per trial arm, limiting within-arm resampling to clusters (hospital units) only would result in a huge number of resamples with identical data (as there are only 9 possible combinations of resampling, with replacement, just 2 units within each of the two trial arms). Therefore, the 2nd step of resampling was necessary. To compensate for the overly conservative 2-step bootstrap CI's, and for potential differences between the two types of units (Internal Medicine and Surgery) within each arm, we then modified slightly the original 2-step bootstrap procedure, as explained below.

The limitation of the 2-step bootstrap in the context of our trial is that a fraction of the 10,000 bootstrap resamples might have resulted in severely un-balanced trial arms, with units resampled, at the 1st step, into I Intervention vs. Control trial arms being non-comparable, for reasons likely different than the intervention. Specifically, in 1,257 (12.6%) of the 10,000 bootstrapped samples combination of units in the intervention and control groups was completely implausible given the trial design, which stratified by type of unit prior to randomization as medicine and surgery have very different outcomes. These combinations are

© 2019 Tamblyn R et al. *JAMA Network Open.*

scenarios 3 and 7 outlined in Table A1. As these 1,257 resamples had systematically different estimates of the intervention effects than the remaining resamples, their inclusion substantially increased the between-resamples variance of our estimates, artificially inflating the resulting 2-step bootstrap CI's. Given (i) this additional variance inflation and (ii) the fact that the 2-step bootstrap (necessary given our trial design, as explained in the previous paragraph) has been shown in simulations to systematically over-estimate the variance[4], we have decided to limit our 2-step bootstrap results to resamples, in which both types of units were resampled in both the intervention and control groups, or internal medicine or surgery were represented in both the intervention and control groups (scenario #1 & #9). The resulting 95% CI are shown in Tables 2 and 3 in the main manuscript. In the following tables A.2a vs A.2b we compare these results with the overly conservative 95% CI's based on all 10,000 resamples. It is evident that restriction to 8,743 resamples did not systematically affect the point estimates of the intervention effect but reduced the width of the 95% CIs. Finally, Table A.2c, at the end of the Appendix, reports the 95% CIs obtained through a standard 1-step bootstrap, in which in each of the 10,000 bootstrap resamples (i) the originally randomized units were retained, and (ii) only individual patients within each unit were resampled, with replacement. It is evident that the simpler 1-step bootstrap (Table A.2c) yields systematically more narrow 95% CI's than our final 2-step bootstrap CI's, based on 8,743 resamples (Table A.2b), demonstrating that our approach accounts for the variance increase induced by clustering within units.

Table A.1; Frequency Distribution of Combinations of Units Allocated to the Intervention and Control Groups in the Bootstrap Samples

Sample unit combinations<br>C=control; I=intervention	**Frequency**	**Percent**	**Cumulative Frequency**	**Cumulative Percent**
**1.** C-Thoracic Surgery, C-Thoracic Surgery, I-Cardiac Surgery, I-Cardiac Surgery	672	6.72	672	6.72
**2.** C-Thoracic Surgery, C-Thoracic Surgery, I-Cardiac Surgery, I-Internal Medicine	1245	12.45	1917	19.17
**3.** C-Thoracic Surgery, C-Thoracic Surgery, I-Internal Medicine, I-Internal Medicine	647	6.47	2564	25.64
**4.** C-Thoracic Surgery, C-Internal Medicine, I-Cardiac Surgery, I-Cardiac Surgery	1239	12.39	3803	38.03
**5.** C-Thoracic Surgery, C-Internal Medicine, I-Cardiac Surgery, I-Internal Medicine	2505	25.05	6308	63.08
**6.** C-Thoracic Surgery, C-Internal Medicine, I-Internal Medicine, I-Internal Medicine	1226	12.26	7534	75.34
**7.** C-Internal Medicine, C-Internal Medicine, I-Cardiac Surgery, I-Cardiac Surgery	610	6.10	8144	81.44
**8.** C-Internal Medicine, C-Internal Medicine, I-Cardiac Surgery, I-Internal Medicine	1226	12.26	9370	93.70
**9.** C-Internal Medicine, C-Internal Medicine, I-Internal Medicine, I-Internal Medicine	630	6.30	10000	100.00

© 2019 Tamblyn R et al. *JAMA Network Open.*

Table A.2a – Mean Odds Ratios and 95% Confidence Intervals, based on 2-step Bootstrap with all 10,000 Bootstrap Resamples

	Overall (N=3,491)	Intervention (N=1,655)	Control (N=1,836)	OR	Empirical 95% CI
	N (%)	N (%)	N (%)		
**Process Outcomes**					
Any Medication Discrepancy	1,466 (42.0)	437 (26.4)	1,029 (56.0)	0.26	0.12-0.82
Error of omission	919 (26.3)	131 (7.9)	788 (42.9)	0.08	0.01-1.21
Therapy duplication	225 (6.4)	39 (2.4)	186 (10.1)	0.04	0.00-0.62
Unintended dose change	742 (21.3)	328 (19.8)	414 (22.5)	0.84	0.48-1.82
**Primary Outcome**					
Adverse Drug Event	149 (4.3)	76 (4.6)	73 (4.0)	0.86	0.33-1.54
Definitely preventable	114 (3.3)	58 (3.5)	56 (3.1)	0.73[4]	0.29-1.33
Probably preventable	30 (0.9)	16 (1.0)	14 (0.8)	1.68	0.15-12.04
Probably/definitely not preventable	5 (0.1)	2 (0.1)	3 (0.2)	-	-
**Secondary Outcomes at 30 Days Post-Discharge**					
ED visits	921 (25.8)	433 (26.2)	488 (26.6)	0.77	0.34-1.51
Readmission to hospital	431 (12.3)	170 (10.3)	261 (14.2)	0.25	0.06-1.31
ED visit, readmission, or death	953 (27.3)	447 (27.0)	506 (27.6)	0.70	0.34-1.27
**Secondary Outcomes at 90 Days Post-Discharge**					
ED visits	1,518 (43.5)	694 (41.9)	824 (45.0)	0.93	0.66-1.27
Readmission to hospital	725 (20.8)	292 (17.6)	433 (23.6)	0.40	0.10-1.90
ED visit, readmission, or death	1,600 (45.8)	728 (44.0)	872 (47.5)	0.86	0.62-1.18

Table A.2b –Mean Bootstrapped Odds Ratios and 95% Confidence Intervals for the 8,743 Bootstrap ReSamples that Excluded Most Unbalanced Combinations (Combinations #3 & 7 in Table A.1)

	Overall (N=3,491)	Intervention (N=1,655)	Control (N=1,836)	OR	Empirical 95% CI
	N (%)	N (%)	N (%)		
**Process Outcomes**					
Any Medication Discrepancy	1,466 (42.0)	437 (26.4)	1,029 (56.0)	0.25	0.12-0.57
Error of omission	919 (26.3)	131 (7.9)	788 (42.9)	0.08	0.02-0.41
Therapy duplication	225 (6.4)	39 (2.4)	186 (10.1)	0.05	0.00-0.34
Unintended dose change	742 (21.3)	328 (19.8)	414 (22.5)	0.84	0.49-1.81
**Primary Outcome**					
Adverse Drug Event	149 (4.3)	76 (4.6)	73 (4.0)	0.86	0.33-1.48
Definitely preventable	114 (3.3)	58 (3.5)	56 (3.1)	0.74	0.28-1.31
Probably preventable	30 (0.9)	16 (1.0)	14 (0.8)	1.66	0.20-12.19
Probably/definitely not preventable	5 (0.1)	2 (0.1)	3 (0.2)	-	-
**Secondary Outcomes at 30 Days Post-Discharge**					
ED visits	921 (25.8)	433 (26.2)	488 (26.6)	0.78	0.36-1.42
Readmission to hospital	431 (12.3)	170 (10.3)	261 (14.2)	0.24	0.06-1.14
ED visit, readmission, or death	953 (27.3)	447 (27.0)	506 (27.6)	0.71	0.34-1.27
**Secondary Outcomes at 90 Days Post-Discharge**					
ED visits	1,518 (43.5)	694 (41.9)	824 (45.0)	0.93	0.70-1.22
Readmission to hospital	725 (20.8)	292 (17.6)	433 (23.6)	0.39	0.11-1.40
ED visit, readmission, or death	1,600 (45.8)	728 (44.0)	872 (47.5)	0.86	0.62-1.18

© 2019 Tamblyn R et al. *JAMA Network Open.*

Table A.2c One-Step Bootstrap Mean Odds Ratios and 95% Confidence Intervals for the 10,000 Bootstrap ReSamples

	Overall (N=3,491)	Intervention (N=1,655)	Control (N=1,836)	OR	Empirical 95% CI
	N (%)	N (%)	N (%)		
**Process Outcomes**					
Any Medication Discrepancy	1,466 (42.0)	437 (26.4)	1,029 (56.0)	0.23	0.19-0.29
Error of omission	919 (26.3)	131 (7.9)	788 (42.9)	0.08	0.05-0.10
Therapy duplication	225 (6.4)	39 (2.4)	186 (10.1)	0.09	0.06-0.19
Unintended dose change	742 (21.3)	328 (19.8)	414 (22.5)	0.75	0.59-0.94
**Primary Outcome**					
Adverse Drug Event	149 (4.3)	76 (4.6)	73 (4.0)	0.97	0.63-1.46
Definitely preventable	114 (3.3)	58 (3.5)	56 (3.1)	0.84	0.52-1.33
Probably preventable	30 (0.9)	16 (1.0)	14 (0.8)	1.44	0.59-3.49
Probably/definitely not preventable	5 (0.1)	2 (0.1)	3 (0.2)	-	-
**Secondary Outcomes at 30 Days Post-Discharge**					
ED visits	921 (25.8)	433 (26.2)	488 (26.6)	0.83	0.67-1.01
Readmission to hospital	431 (12.3)	170 (10.3)	261 (14.2)	0.22	0.16-0.29
ED visit, readmission, or death	953 (27.3)	447 (27.0)	506 (27.6)	0.75	0.61-0.91
**Secondary Outcomes at 90 Days Post-Discharge**					
ED visits	1,518 (43.5)	694 (41.9)	824 (45.0)	0.94	0.78-1.14
Readmission to hospital	725 (20.8)	292 (17.6)	433 (23.6)	0.37	0.29-0.47
ED visit, readmission, or death	1,600 (45.8)	728 (44.0)	872 (47.5)	0.87	0.72-1.05

© 2019 Tamblyn R et al. *JAMA Network Open.*

eFigure 1. Screenshot of the RightRx User Interface at Discharge

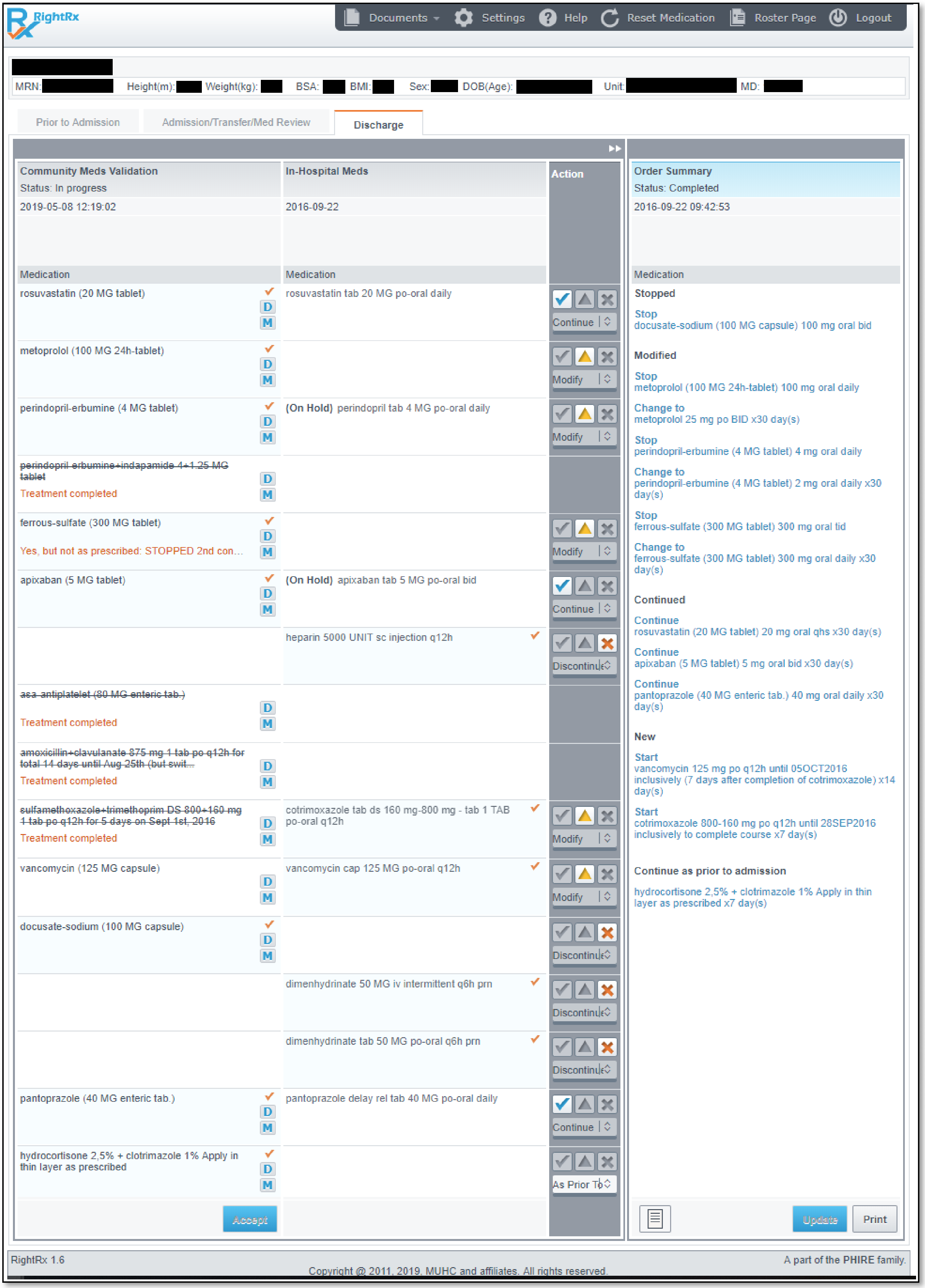

© 2019 Tamblyn R et al. *JAMA Network Open.*

Footnotes eFigure1

1. Drugs were grouped by therapeutic class based on the American Hospital Formulary Classification system and ordered by clinical importance as follows: cardiovascular drugs, followed by anti-coagulants, diuretics, hormones and substitutes (e.g. corticosteroids, insulin), anti-infectives, anti-neoplastics, etc and ending with vitamins and diagnostic agents.
2. By clicking on “D”, the user would have details about the specific drug prescribed including trade name, prescribing physician and telephone number, dispensing pharmacy and telephone number, data entered during community drug list validation including adherence to the medication and reason(s) if applicable for non-adherence, validation of the community drug and the source of validation.
3. By clicking on “M”, the user could view the drug monograph.
4. The action bar has four options: “√”: continue this medication as prescribed, “Δ”: modify this medication (e.g. change in medication, dose or route), “x”: stop this medication, “continue as prior to admission”: continue as prescribed in the community. Each action would appear in the order summary (far right panel) grouped by type of action starting with “stopped medications”, “modified medications” continued medications”, and medications that were to be continued by the community-based prescribing physician. Medications in the “continue as previous bin” would list the community-based prescribing physician and pharmacy and would provide an emergency supply for 7 days to be filled only if the patient was unable to obtain the respective medication from their community-based provider (see e-figure 2). The order when finalized could be printed and signed by the prescribing physician.

© 2019 Tamblyn R et al. *JAMA Network Open.*

eFigure 2. Discharge Prescription

Centre universitaire de santé McGill | McGill University Health Centre | RxSécuritaire

[x] HGM/MGH [ ] HRV/RVH [ ] HME/MCH
[ ] HNM/MNH [ ] ITM/MCI [ ] Lachine

FMU-3082

Nom patient / Patient's Name [redacted]
MRN / MRN [redacted]
#Ass. Maladie / Medicare# [redacted]
Date de naissance / Date of Birth [redacted]

Prescription au congé | Discharge prescription

Date: [redacted] (AAYY/MM/JD) [redacted] (Heure/Time) Service: [redacted]

Poids/Weight: [redacted] Allergies: [redacted] CrCl(mL/min): [redacted]

Médicaments arrêtés | Stopped Medications:

1/10 **STOP** docusate-sodium (100 MG capsule) 100 mg oral bid
Reason for stop: No longer needed
Notes: Started Sept 1st, 2016

Médicaments modifiés | Modified Medications:

		Quantity	Renewals
2/10	**STOP** metoprolol (100 MG 24h-tablet) 100 mg oral daily		
	**CHANGE TO** metoprolol 25 mg po BID x30 day(s)<br>**Reason for change:** Achieve lowest dose		3
3/10	**STOP** perindopril (4 MG tablet) 4 mg oral daily		
	**CHANGE TO** perindopril (4 MG tablet) 2 mg oral daily x30 day(s)<br>**Reason for change:** Taper dose		3
4/10	**STOP** ferrous-sulfate (300 MG tablet) 300 mg oral tid		
	**CHANGE TO** ferrous-sulfate (300 MG tablet) 300 mg oral daily x30 day(s)<br>**Reason for change:** Taper dose<br>**Notes:** Started on Sept 7th, 2016		3

Médicaments à poursuivre | Continued Medications:

		Quantity	Renewals
5/10	**CONTINUE** rosuvastatin (20 MG tablet) 20 mg oral qhs x30 day(s)		3
6/10	**CONTINUE** apixaban (5 MG tablet) 5 mg oral bid x30 day(s)<br>**Notes:** CODE CV155		3
7/10	**CONTINUE** pantoprazole (40 MG enteric tab.) 40 mg oral daily x30 day(s)		3

Nouveaux médicaments | New Medications:

		Quantity	Renewals
8/10	**START** vancomycin 125 mg po q12h until 05OCT2016 inclusively (7 days after completion of cotrimoxazole) x14 day(s)		0
9/10	**START** cotrimoxazole 800-160 mg po q12h until 28SEP2016 inclusively to complete course x7 day(s)		0

Signature du médecin | Physician's signature

[redacted]
Nom en lettres moulées | Name in print

[redacted]
N° permis | License N°

Numéro de téléphone de l'unité de soins au CUSM | Telephone number of the discharging unit at the MUHC:

[redacted]
Numéro de téléphone | Telephone number

[redacted]
Ext.

Cette prescription a été rédigée lorsque le patient a reçu son congé du CUSM. Veuillez adresser toute question concernant ces médicaments, et toute demande de renouvellement au médecin traitant de ce patient.
For problems or renewals concerning any medications within this discharge description, please contact the patient's primary care physician.

Int. Ref.# [redacted]

Page 1/2

© 2019 Tamblyn R et al. *JAMA Network Open.*

Centre universitaire de santé McGill | McGill University Health Centre | RxSécuritaire

☒HGM/MGH ☐HRV/RVH ☐HME/MCH
☐HNM/MNH ☐ITM/MCI ☐Lachine

* F M U - 3 0 8 2 *

Nom patient / Patient's Name [redacted]
MRN / MRN [redacted]
#Ass. Maladie / Medicare# [redacted]
Date de naissance / Date of Birth [redacted]

Prescription au congé | Discharge prescription

Date: [redacted] AAYY/MM/JD [redacted] Heure/Time   Service: [redacted]

Poids/Weight: [redacted]   Allergies: [redacted]   CrCl(mL/min): [redacted]

Médicaments pris avant l'hospitalisation | Medications prior to admission:

The following medications were prescribed **PRIOR TO ADMISSION. PLEASE CONTACT LAST PRESCRIBER TO AUTHORIZE CONTINUED USE** according to the treatment plan of the original prescribing physician. An emergency supply is authorized only if patient does not have medication available at discharge

	Quantity	Last Prescriber	Last Pharmacy
10/10 **CONTINUE** hydrocortisone 2,5% + clotrimazole 1% Apply in thin layer as prescribed x7 day(s)			

Signature du médecin | Physician's signature

[redacted] Nom en lettres moulées | Name in print

[redacted] N° permis | License N°

Numéro de téléphone de l'unité de soins au CUSM | Telephone number of the discharging unit at the MUHC:

[redacted] Numéro de téléphone | Telephone number

[redacted] Ext.

Cette prescription a été rédigée lorsque le patient a reçu son congé du CUSM. Veuillez adresser toute question concernant ces médicaments, et toute demande de renouvellement au médecin traitant de ce patient.
For problems or renewals concerning any medications within this discharge description, please contact the patient's primary care physician.

Int. Ref.# [redacted]

© 2019 Tamblyn R et al. *JAMA Network Open.*

eFigure 3. ADE Adjudication Forms

Assessment Of Adverse Drug Events

Adding new MRN **XXXXX**

**MRN**	XXXXX
**Please indicate your reviewer status:** * must provide value	▼

Index Hospitalization Information.

**Date of Admission**	Today M-D-Y
**Date of Discharge**	Today M-D-Y

Main diagnoses at admission:

Taken from Med-Echo (Quebec's hospital discharge summary database) and RAMQ (Quebec's healthcare claims database).

This field is prepopulated with information about the patient's diagnoses at admission.

Expand

All documented diagnoses at discharge:

Taken from Med-Echo (Quebec's hospital discharge summary database).

Diagnoses for hospitalization + comorbidities.

This field is prepopulated with information about the patient's diagnoses at discharge.

Expand

Comorbidities in the Year Prior:

Taken from RAMQ (Quebec's healthcare claims database).

This field is prepopulated with information about the patient's comorbidities in the year prior to admission.

Expand

© 2019 Tamblyn R et al. *JAMA Network Open.*

Discharge Prescription:

This field is prepopulated with a list of the medications prescribed at discharge.

Expand

Dispensed Medications 30 Days Post-Discharge:
Taken from RAMQ (Quebec's healthcare claims database).

This field is prepopulated with a list of medications dispensed to the patient in the 30 days following hospital discharge.

Expand

RAMQ Medical Services 30 Days Post-Discharge
Please click here for a list of definitions for establishment numbers included below (ex.0XXX1=outpatient clinic).

This field is prepopulated with a list of the medical services the patient received in the 30 days following hospital discharge.

Expand

Identified Adverse Drug Event(s)

Source of Problem(s) Identified:

- [x] Interview
- [x] ER Visit
- [x] Readmission

Patient self-reported problem from the interview:

If applicable, this field is prepopulated with the problem identified in the patient interview that is to be adjudicated.

Expand

30-day post-discharge ER visit diagnoses:

If applicable, this field is prepopulated with the post-discharge ED visit diagnosis that is to be adjudicated.

Expand

© 2019 Tamblyn R et al. *JAMA Network Open.*

30-day post-discharge readmission diagnoses:

If applicable, this field is prepopulated with the post-discharge hospitalization diagnosis that is to be adjudicated.

Expand

Assessment of Identified Adverse Drug Event(s).

Please use your professional experience and information provided above regarding index hospitalization, as well as drug dispensation and medical services usage in the 30 days post-discharge to answer the following questions.

How likely was at least one of the above identified problem(s) an adverse drug event (ADE)?

* must provide value

Very unlikely: 0-15% | Possible: 16-49%; Probable: 50-84% | Very likely: 85-100%

67

reset

What do you think is the drug(s) responsible for this ADE? And please indicate the types of problems for EACH DRUG that may have lead to this ADE in brackets.

(Ex. WARFARIN (Patient Non-adherence))

* must provide value

Expand

How preventable could this problem have been?

* must provide value

Definitely NOT preventable: 0-15% | Probably not preventable: 16-49%; Probably preventable: 50-84% | Definitely preventable: 85-100%

50

reset

© 2019 Tamblyn R et al. *JAMA Network Open.*

Could the severity of this ADE or potential ADE have been significantly reduced by earlier detection or intervention?

* must provide value

Severity definitely could NOT have been reduced: 0-15%	Probably could not have been reduced: 16-49%; Probably could have been reduced: 50-84%	Severity definitely could have been reduced: 85-100%

[slider] 50

reset

Form Status

Complete? Incomplete ▼

Lock this record for this form?

If locked, no user will be able to edit this record on this form until someone with Lock/Unlock privileges unlocks it.

☐ Lock

Save & Exit Form | Save & Stay

-- Cancel --

© 2019 Tamblyn R et al. *JAMA Network Open.*

eReferences

1. Kahan BC, Forbes G, Ali Y, et al. Increased risk of type I errors in cluster randomised trials with small or medium numbers of clusters: a review, reanalysis, and simulation study. *Trials.* 2016;17(1):438-438.
2. Li P, Redden DT. Comparing denominator degrees of freedom approximations for the generalized linear mixed model in analyzing binary outcome in small sample cluster-randomized trials. *BMC medical research methodology.* 2015;15:38-38.
3. Leyrat C, Morgan KE, Leurent B, Kahan BC. Cluster randomized trials with a small number of clusters: which analyses should be used? *International Journal of Epidemiology.* 2018;47(3):1012-1012.
4. Xiao Y, Abrahamowicz M. Bootstrap-based methods for estimating standard errors in Cox's regression analyses of clustered event times. *Statistics in medicine.* 2010;29(7-8):915-923.

© 2019 Tamblyn R et al. *JAMA Network Open.*
