# Supplementary material for: Effect of an Electronic Medication Reconciliation Intervention on Adverse Drug Events: A Cluster Randomized Trial
Source: JAMA Netw Open. 2019 Sep 20;2(9):e1910756. doi: 10.1001/jamanetworkopen.2019.10756 (PMC6755531; doi:10.1001/jamanetworkopen.2019.10756)
Supplement: Supplement 3. — Data Sharing Statement [file jamanetwopen-2-e1910756-s003.pdf]

Data Sharing Statement

Tamblyn. Effect of an Electronic Medication Reconciliation Intervention on Adverse Drug Events. *JAMA Netw Open*. Published September 20, 2019. 10.1001/jamanetworkopen.2019.10756

Data
Data available: No

Additional Information
Explanation for why data not available: Not permitted by ethics committee
